# Heterogeneity-driven phenotypic plasticity and treatment response in branched-organoid models of pancreatic ductal adenocarcinoma

**DOI:** 10.1038/s41551-024-01273-9

**Published:** 2024-12-10

**Authors:** Aristeidis Papargyriou, Mulham Najajreh, David P. Cook, Carlo H. Maurer, Stefanie Bärthel, Hendrik A. Messal, Sakthi K. Ravichandran, Till Richter, Moritz Knolle, Thomas Metzler, Akul R. Shastri, Rupert Öllinger, Jacob Jasper, Laura Schmidleitner, Surui Wang, Christian Schneeweis, Hellen Ishikawa-Ankerhold, Thomas Engleitner, Laura Mataite, Mariia Semina, Hussein Trabulssi, Sebastian Lange, Aashreya Ravichandra, Maximilian Schuster, Sebastian Mueller, Katja Peschke, Arlett Schäfer, Sophie Dobiasch, Stephanie E. Combs, Roland M. Schmid, Andreas R. Bausch, Rickmer Braren, Irina Heid, Christina H. Scheel, Günter Schneider, Anja Zeigerer, Malte D. Luecken, Katja Steiger, Georgios Kaissis, Jacco van Rheenen, Fabian J. Theis, Dieter Saur, Roland Rad, Maximilian Reichert

**Affiliations:** 1 Translational Pancreatic Cancer Research Center, Klinik und Poliklinik für Innere Medizin II, Klinikum rechts der Isar, Technical University of Munich, München, Germany; 2 Klinik und Poliklinik für Innere Medizin II, Klinikum rechts der Isar, Technical University of Munich, München, Germany; 3 Center for Functional Protein Assemblies, Technical University of Munich, Garching, Germany; 4 Center for Organoid Systems (COS), Technical University of Munich, Garching, Germany; 5 Bavarian Cancer Research Center (BZKF), Munich, Germany; 6 Institute of Stem Cell Research, Helmholtz Center Munich, Neuherberg, Germany; 7 Department of Cellular and Molecular Medicine, Faculty of Medicine, University of Ottawa, Ottawa, Ontario Canada; 8 Cancer Therapeutics Program, Ottawa Hospital Research Institute, Ottawa, Ontario Canada; 9 Center for Translational Cancer Research (TranslaTUM), School of Medicine, Technical University of Munich, Munich, Germany; 10 Chair for Translational Cancer Research and Institute of Experimental Cancer Therapy, Klinikum rechts der Isar, School of Medicine, Technical University of Munich, Munich, Germany; 11 Division of Translational Cancer Research, German Cancer Research Center (DKFZ) and German Cancer Consortium (DKTK), Heidelberg, Germany; 12 Division of Molecular Pathology, Oncode Institute, The Netherlands Cancer Institute, Amsterdam, the Netherlands; 13 Institute of Computational Biology, Helmholtz Center Munich, Neuherberg, Germany; 14 Department of Mathematics, School of Computing, Information and Technology, Technical University of Munich, Munich, Germany; 15 Institute of Diagnostic and Interventional Radiology, Klinikum rechts der Isar München, Technical University of Munich, Munich, Germany; 16 Artificial Intelligence in Medicine and Healthcare, Technical University of Munich, Munich, Germany; 17 Comparative Experimental Pathology, Institut für Allgemeine Pathologie und Pathologische Anatomie, School of Medicine, Technical University of Munich, Munich, Germany; 18 Institute of Molecular Oncology and Functional Genomics, School of Medicine, Technical University of Munich, Munich, Germany; 19 Institute for Diabetes and Cancer, Helmholtz Center Munich, Neuherberg, Germany; 20 Joint Heidelberg-IDC Translational Diabetes Program, Heidelberg University, Heidelberg, Germany; 21 German Center for Diabetes Research (DZD), Neuherberg, Germany; 22 Department of Medicine I, University Hospital of the Ludwig-Maximilians-University Munich, Munich, Germany; 23 Department of Radiation Oncology, Technical University of Munich, Munich, Germany; 24 Institute of Radiation Medicine (IRM), Helmholtz Zentrum München, Neuherberg, Germany; 25 Lehrstuhl für Zell Biophysik E27, Physik Department, Technische Universität München, Garching, Germany; 26 Department of Dermatology, Ruhr-University Bochum, Bochum, Germany; 27 Department of General, Visceral and Pediatric Surgery, University Medical Center Göttingen, Göttingen, Germany; 28 European Center for Angioscience (ECAS), Medical Faculty Mannheim, Heidelberg University, Mannheim, Germany; 29 Institute of Lung Health and Immunity (LHI), Helmholtz Munich, Comprehensive Pneumology Center (CPC-M), München, Germany; 30 Institute for Machine Learning in Biomedical Imaging, Helmholtz Zentrum München, München, Germany; 31 Department of Computing, Imperial College London, London, UK; 32 Munich Center for Machine Learning (MCML), München, Germany; 33 School of Computation, Information and Technology, Technische Universität München, München, Germany; 34 Cellular Genetics Programme, Wellcome Sanger Institute, Hinxton, Cambridge UK; 35 German Cancer Consortium (DKTK), partner site Munich, Munich, Germany; 36 Munich Institute of Biomedical Engineering (MIBE), Technical University of Munich, Munich, Germany

**Keywords:** Cancer stem cells, Tumour heterogeneity, Epithelial-mesenchymal transition, Pancreatic cancer, Targeted therapies

## Abstract

In patients with pancreatic ductal adenocarcinoma (PDAC), intratumoural and intertumoural heterogeneity increases chemoresistance and mortality rates. However, such morphological and phenotypic diversities are not typically captured by organoid models of PDAC. Here we show that branched organoids embedded in collagen gels can recapitulate the phenotypic landscape seen in murine and human PDAC, that the pronounced molecular and morphological intratumoural and intertumoural heterogeneity of organoids is governed by defined transcriptional programmes (notably, epithelial-to-mesenchymal plasticity), and that different organoid phenotypes represent distinct tumour-cell states with unique biological features in vivo. We also show that phenotype-specific therapeutic vulnerabilities and modes of treatment-induced phenotype reprogramming can be captured in phenotypic heterogeneity maps. Our methodology and analyses of tumour-cell heterogeneity in PDAC may guide the development of phenotype-targeted treatment strategies.

## Abstract (web summary)

Branched organoids embedded in collagen gels can recapitulate the phenotypic landscape and pronounced molecular and morphological heterogeneity of pancreatic ductal adenocarcinoma.

## Main

Pancreatic cancer is expected to surpass colorectal cancer as the second leading cause of cancer-related deaths by 2025, trailing only lung cancer^1^. PDAC is characterized by a pronounced intertumoural and intratumoural heterogeneity^2^. While numerous studies have identified distinct molecular PDAC subtypes on a transcriptional level^3,4^, it is likely that these subtypes are not static and present a continuum^5^. As several of these subtypes co-exist in a single tumour, diverse treatment responses and patient outcomes are observed^6–8^. It has been demonstrated that in many cancer types, intratumoural heterogeneity (ITH) is achieved by existing or emerging subclonal driver mutations^9^. PDAC, however, displays multiple clonal driver mutations at its evolutionary root and the ITH involved in PDAC progression and treatment resistance is regulated preferentially by non-mutational epigenetic reprogramming, specifically phenotypic plasticity^2,10,11^. Indeed, the ability of cancer cells to overcome the physiologically restricted phenotypic plasticity is a critical trait to deviate from the state of terminal differentiation^12^ and retain adaptability^11^, making this ‘unlocking’ of phenotypic plasticity an emerging hallmark of cancer^13^. To identify novel therapeutic strategies to inhibit these characteristics by state-gating and state-targeting strategies, it is critical to develop biomarkers to functionalize ITH in PDAC^11^.

We have recently developed a branching organoid model system which recapitulates the tubular morphology of PDAC^14^. Here we demonstrate that, in contrast to other current organoid model systems, the branched-organoid model can display the phenotypic diversity of distinct PDAC subtypes as well as within individual tumours and, therefore, captures intratumoural and intertumoural heterogeneity. We generated a morphological and transcriptional phenotypic landscape of tumour heterogeneity of branched organoids derived from defined transcriptional PDAC subtypes and patient-derived organoids. Importantly, morphologically distinct families of organoids display unique functional properties associated with key biological features of PDAC, including tumour cell states, epithelial-to-mesenchymal transition (EMT) plasticity, metastatic capacities and responses to cytotoxic and targeted therapy. Lastly, we implemented our organoid model system in combination with comprehensive image-based phenotypic analysis and pharmacotyping to identify state-gating and state-targeting monotherapies and combinatorial treatments.

## Results

### PDAC molecular subtypes give rise to morphologically distinct organoids

**Fig. 1:**
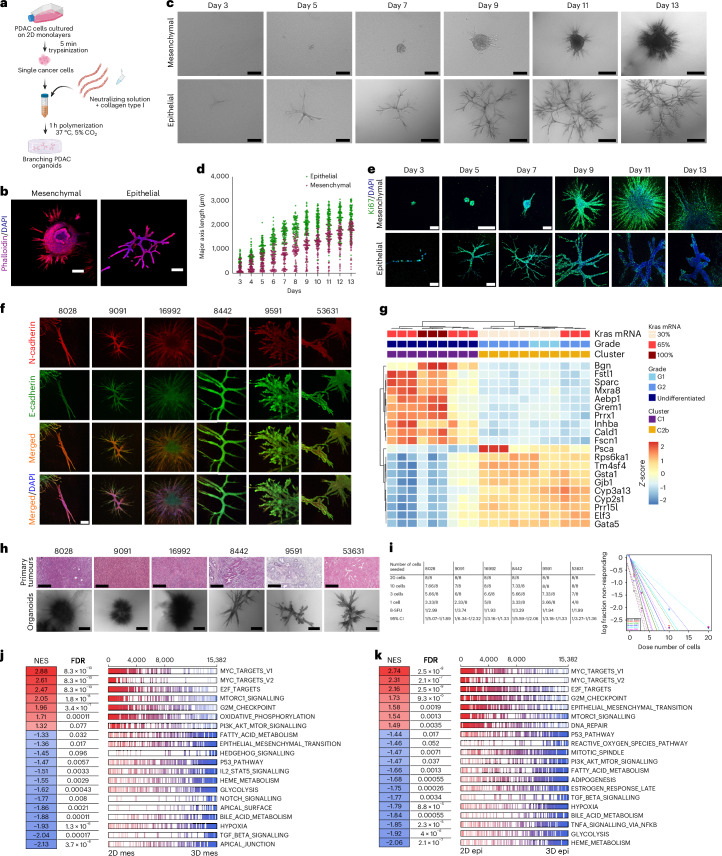
PDAC subtypes give rise to morphologically distinct branching organoids. **a**, Schematic representation of the preparation of PDAC organoid cultures in floating collagen gels. Image created with BioRender.com. **b**, Confocal IF imaging of the organoid cytoskeleton (purple) and DAPI (blue) for epithelial and mesenchymal organoids. Images are maximum projections generated with Imaris. Scale bars, 200 µm (for mesenchymal), 500 µm (for epithelial organoids). **c**, Daily imaging of single-cell-derived organoids from the epithelial line (ID: 9591) and the mesenchymal line (ID: 16992) over the course of 13 days of development (here displaying Days 3–13). Scale bars, mesenchymal: 200 μm (Days 3–9), 500 μm (afterwards); epithelial: 200 μm (Days 3–5), 500 μm (afterwards). **d**, Major axis length of the organoid development of *n* = 1,099 epithelial (from the mouse line ID: 9591, 3 individual experiments) and *n* = 904 mesenchymal (from the mouse line ID: 16992, 3 individual experiments) organoids. Plot presents mean ± s.e.m. **e**, Confocal IF imaging of the proliferation marker Ki67 (green) and DAPI (blue) for epithelial (from the mouse line ID: 9591, 3 individual experiments) and mesenchymal (from the mouse line ID: 16992, 3 individual experiments) organoids. Scale bars, 100 µm (Day 3) and 200 µm (Day 5 onwards) for mesenchymal panel; 50 µm (Day 3) and 200 µm (Day 5 onwards) for epithelial panel. **f**, Confocal IF imaging of the epithelial marker E-cadherin (green), the mesenchymal marker N-cadherin (red) and DAPI (blue) for mesenchymal (*n* = 3 independent mouse lines; IDs: 8028, 9091, 16992) and epithelial (*n* = 3 independent mouse lines; IDs: 8442, 9591, 53631) organoids. Scale bars, 200 µm. **g**, Hierarchical clustering of RNA sequencing data from epithelial and mesenchymal 3D organoids derived from different Kras^G12D^ background mice. Tumour grading, Kras mRNA levels retrieved from a previous study^15^. Heat map of the leading-edge genes for both clusters. **h**, H&E staining of the primary tumours and corresponding brightfield organoid morphologies (*n* = 3 mouse lines for mesenchymal, *n* = 3 mouse lines for epithelial). Scale bars, 200 µm (H&E), 500 µm (organoids). **i**, ELDA of epithelial and mesenchymal organoids (left) and plot of the log fraction of non-responding wells (without organoids) versus the number of seeded cells (right). **j**,**k**, GSEA comparing 2D mesenchymal and 3D mesenchymal organoids (**j**) and 2D epithelial and 3D epithelial organoids (**k**). Every bar represents individual genes for the given gene set. Source data

**Extended Data Fig. 1:**
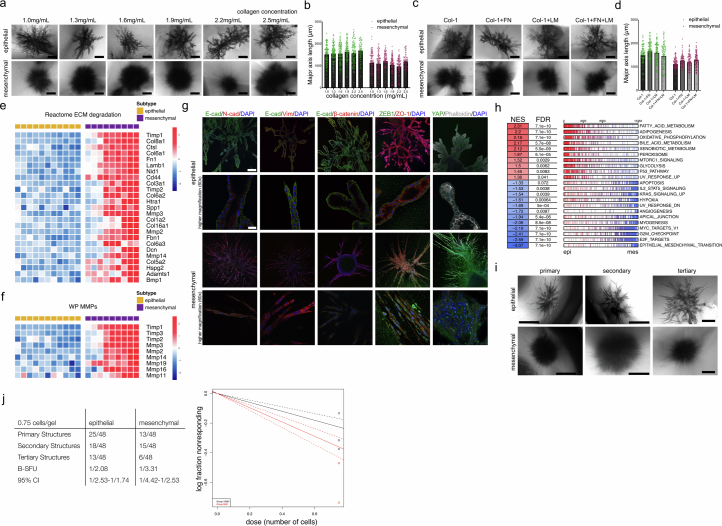
Characterization of epithelial and mesenchymal branching in PDAC organoids. **a** Organoid morphologies of epithelial (upper panel mouse line ID: 9591, from 3 independent experiments-exception is the concentration 2.5 mg/mL with only 2 independent replicates included) and mesenchymal (lower panel mouse line ID 16992, from 3 independent experiments-exception is the concentration 2.5 mg/mL with only 2 independent replicates included) organoids grown in various collagen concentrations from 1.0 mg/mL up to 2.5 mg/mL, Scale bars, 500 μm. **b** Quantification of the organoids major axis length in the different collagen concentrations (n = 612 for the epithelial and n = 563 for the mesenchymal), graph represents mean±sem. **c** Organoid morphologies for epithelial (upper panel mouse line ID: 9591, 3 independent experiments) and mesenchymal (lower panel mouse line ID: 16992, 3 independent experiments) organoids grown in collagen Type I gels with the addition of the ECM proteins fibronectin (FN, concentration 40 µg/mL), laminin (LM, concentration 40 µg/mL) or the combination (FN + LM), Scale bars, 500 μm. **d** Quantification of the organoids major axis length in the different collagen Type I matrices with the addition of FN, LM or both (n = 378 organoids for the epithelial and n = 388 organoids for the mesenchymal), graph represents mean±sem. **e-f** Heatmaps of most up and down-regulated genes between epithelial and mesenchymal organoids for the “Reactome ECM degradation” and the “WP MMPs”. **g** Confocal IF imaging of an EMT panel, first column: E-cadherin (green) /N-cadherin (red) /DAPI (blue), second column: E-cadherin (green) /Vimentin (red) /DAPI (blue), third column: E-cadherin (green) /β-catenin (red) /DAPI (blue), fourth column: ZEB-1 (green) /ZO-1 (red) /DAPI (blue) and fifth column: YAP (green) / phalloidin (white) /DAPI (blue) for epithelial (top) and mesenchymal organoids (bottom), scale bars upper panel (10x magnification)= 200 μm, lower panel-higher magnifications (60x)= 30 μm. Images on the second and fourth row are taken on a 60x objective (higher magnification) of their respective upper panel or from the similar organoid phenotypes. **h** Gene set enrichment analysis (GSEA) comparing epithelial and mesenchymal 3D organoid cultures, NES= normalized enrichment scores, FDR= false discovery rate. Every bar represents individual genes for the given gene set. **i** Morphologies of epithelial and mesenchymal organoids in primary, secondary and tertiary structures. Scale bars primary structures= 1000 μm, secondary 1000 for the epithelial and 500 μm for the mesenchymal and tertiary= 500 μm. **j** Extreme limiting dilution analysis (ELDA) and log plot of nonresponding wells vs. cell dosage for the primary, secondary, and tertiary structures of epithelial and mesenchymal organoids. Source data

A collection of primary PDAC cancer cells from genetically engineered pancreatic cancer mouse models (Ptf1a^Cre/+^;Kras^G12D/+^;KC) were previously generated and clustered according to their transcriptional subtypes^15^. Transcriptionally as well as morphologically, these PDAC cells cultured under standard two-dimensional (2D) culture conditions on plastic dishes display typical epithelial or mesenchymal features depending on the respective *Kras*^*G12D*^ gene dosages^15^. To expand on this dichotomous classification of epithelial versus mesenchymal features, we hypothesized that by implementing the branching organoid model system^14^, we could generate phenotypically diverse organoids to capture and functionalize the entire EMT continuum of PDAC subtypes. To this end, PDAC cells from distinct transcriptional clusters were embedded at clonal densities inside a 3D floating collagen type-I matrix^14,16^ (Fig. 1a) and allowed to grow for 13 days to generate complex multicellular branched organotypic structures (Fig. 1b). Over the course of 13 days of development, epithelial tumour organoids formed multiple main and sub-branches akin to a complex ductal network (Fig. 1c). After Day 10, epithelial organoids reached a maximum size, illustrated by the major axis length (Fig. 1d). At this point, they stopped expanding and were rather fully mature, with the formation of terminal end buds and a seamless lumen connecting the entire organoid body (Fig. 1b,c). In contrast, mesenchymal tumour organoids appeared much more compact, with a main organoid body densely packed with cells. On Day 7, mesenchymal organoids reached a critical mass and started invading the collagen matrix via protrusions (Fig. 1c). Eventually, mesenchymal organoids grew in an invasive manner with a main cellular core and long branches, continuously invading into the matrix and following a linear type of growth (Fig. 1c,e). The extracellular matrix (ECM) plays a key role during pancreas development and morphogenesis^17^ as well as during PDAC progression, acting both as a natural scaffold and having functional roles as signal transducer via biochemical and biomechanical cues^18^. Fibrillar collagens (COL1A1, COL1A2, COL3A1) are the most abundant proteins found both in normal pancreas and in pancreatic cancer^19^. These collagens are of key importance together with other matricellular proteins, fibronectin and laminins, for the adhesion of PDAC cells supporting organoid formation in polyethylene glycol (PEG) gels^20^. We sought to investigate whether different collagen concentrations or different core-matrisome proteins (fibronectin, laminin) exert an effect on organoid growth and morphogenesis. We tested collagen concentrations ranging from 1.0 mg ml^−1^ (relatively soft), previously established 1.3 mg ml^−1^ (ref. ^14^) to 2.5 mg ml^−1^ (stiff). Organoids grown at indicated collagen concentrations displayed neither notable morphological differences (Extended Data Fig. 1a) nor substantial changes in growth, except for a slight increase in the major axis length from 1,550.74 µm in 1.3 mg ml^−1^ to 1,686.19 µm (mean) in the 2.5 mg ml^−1^ collagen concentration for the epithelial, and from 1,096.08 µm (mean) in 1.3 mg ml^−1^ to 1,192.15 µm in the 2.5 mg ml^−1^ concentration for the mesenchymal organoids (Extended Data Fig. 1b). Similarly, the addition of the glycoproteins fibronectin (FN) and/or laminin (LM) did not foster any qualitative morphological changes, only exerting minor changes in organoid size (Extended Data Fig. 1c). In detail, the addition of FN led to an increased mean major axis length by 142.73 µm in the epithelial and 144.75 µm in mesenchymal organoids. The addition of laminin (LM) increased the major axis length by 85.65 µm in the epithelial and by 65.12 µm in the mesenchymal organoids (Extended Data Fig. 1d). Regarding matrix remodelling, mesenchymal organoids display high expression of matrix metalloproteinases mediating increased ECM degradation compared with epithelial organoids (Extended Data Fig. 1e,f).

These distinct organoid morphologies from epithelial and mesenchymal PDAC subtypes maintain the distribution of expression of key EMT markers, such as E-cadherin, Vimentin, N-cadherin, ZO-1, ZEB1 and β-catenin, matching the transcriptional subtypes (Fig. 1f and Extended Data Fig. 1g).

Next, we performed transcriptomic profiling of branching organoids to test whether these organoids retain the transcriptional subtypes of their parental PDAC cell lines. Indeed, two separate clusters could be identified, with enrichment of signatures such as fatty acid metabolism, OXPHOS, P53 signalling and glycolysis in the epithelial subcluster, and typical basal-like signatures such as EMT, E2F targets, Myc targets, Kras signalling and hypoxia in the mesenchymal subcluster (Fig. 1g and Extended Data Fig. 1h). Importantly, on a morphological level, branching organoids recapitulated the in vivo tumour architecture to a remarkable degree when comparing the primary tumours (glandular vs non-glandular) to the organoid structures (Fig. 1h). Furthermore, these single-cell-derived branched organoids retained their branching ability and the morphological features of the parental organoid line for a series of passages, with high potencies of generating new branching organoids of 1/2.08 cells for the epithelial and 1/3.31 for the mesenchymal organoids (Fig. 1i and Extended Data Fig. 1i,j). Given the complex phenotype of branching organoids in 3D, we next compared gene-expression profiles of epithelial and mesenchymal organoids to corresponding parental 2D PDAC cells (3D vs 2D culture conditions). In 2D monocultures, we found enriched proliferation signatures such as E2F targets (normalized enrichment score (NES): 2.47 for the mesenchymal and 2.16 for the epithelial) and G2M checkpoint (NES: 1.96 for the mesenchymal and 1.73 for the epithelial). In contrast, 3D mesenchymal organoids were enriched for signatures of Hedgehog (NES: 1.45) and Notch signalling (NES: 1.77). Interestingly, one of the most profound differences in enriched gene signatures in 3D vs 2D in both PDAC subtypes is the TGFβ signalling pathway (NES: 2.04 for the mesenchymal and 1.77 for the epithelial organoids) (Fig. 1j,k).

Altogether, the data show that branching PDAC organoids retain their EMT identity and establish distinct morphologies based on the parental PDAC subtype. Moreover, branching PDAC organoids activate distinct transcriptional programmes involving fundamental developmental and morphogenesis signalling pathways.

### Canonical TGFβ signalling is required for branch formation in PDAC organoids

On the basis of our gene-expression profiling and identification of differentially regulated pathways between PDAC cells cultured in 3D or 2D (Fig. 1j,k), we sought to functionally investigate pathways directing branching morphogenesis in PDAC organoids. To this end, we performed inhibitory and stimulatory experiments, focusing on pivotal developmental pathways enriched in organoid cultures such as EGFR, Wnt/β-catenin, Hedgehog, Notch and TGFβ, and examined their effects on organoid morphology (*n* = 1,918 organoids).

**Fig. 2:**
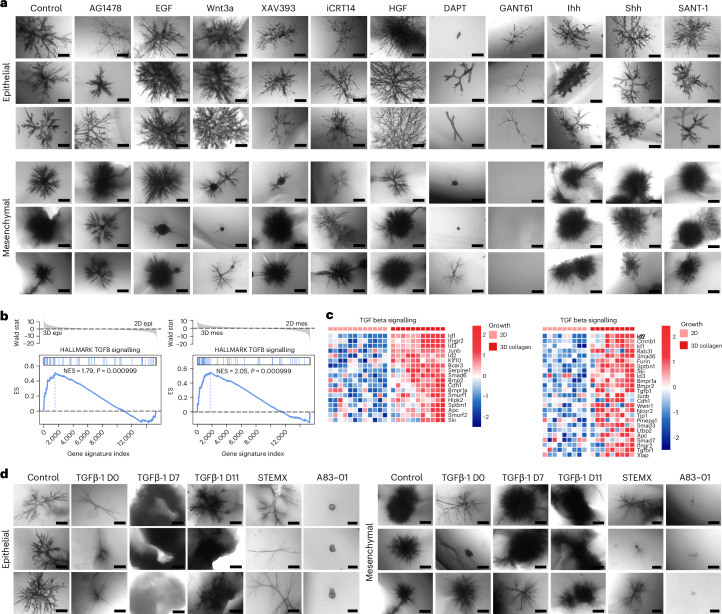
Canonical TGFβ signalling is essential for the formation of branching PDAC organoids for both epithelial and mesenchymal subtypes. **a**, Morphological effect of major developmental pathways: EGFR by AG1478 (EGFR inhibitor) and EGF, Wnt/β-catenin by Wnt3a, XAV393 (Tankyrase inhibitor), iCRT14 (β-catenin-Tcf inhibitor), Notch (γ-secretase inhibitor), HGF, and Hedgehog by Ihh, Shh, GANT61 (GLI antagonist) and SANT-1 (Smo antagonist) on epithelial (*n* = 1,045 organoids, line ID: 9591, 3 individual experiments) and mesenchymal organoids (*n* = 873 organoids, line ID: 16992, 3 individual experiments). Treatments were performed with 10 µM AG1478, 5 ng EGF, 100 ng Wnt3a, 5 µM XAV939, 5 µM iCRT14, 5 ng HGF, 10 µM DAPT, 10 µM GANT61, 100 ng Ihh, 100 ng Shh, 2 µM Sant-1. Scale bars, 500 µm. **b**, GSEA of epithelial (*n* = 3 mouse lines, from 3 individual experiments) and mesenchymal (*n* = 3 mouse lines, from 3 individual experiments) 2D monolayers and 3D organoids for the TGFβ pathway. **c**, Heat maps of most up and downregulated genes between 2D monolayers and 3D cells for epithelial and mesenchymal organoids (from left to right). **d**, Monotreatments of Kras^G12D^ epithelial (*n* = 468 organoids, line ID: 9591, 3 individual experiments) or mesenchymal (*n* = 618 organoids, line ID:16992, 3 individual experiments) organoids with 5 ng of TGFβ-1; treatment administration from Day 0, Day 7 or Day 11 after seeding, 1× StemXVivo EMT-inducing media supplement (termed STEMX) treatment from Day 0, or 5 µM TGFβ-RI inhibitor A83-01 treatment from Day 0. All organoids were imaged on Day 13. Scale bars, epithelial organoids: 200 µm (all A83-01), 500 µm (all others); mesenchymal organoids: 200 µm (all STEMX and the bottom A83-01), 500 µm (all others).

While manipulation of EGFR and Wnt/β-catenin did not substantially alter the organoids’ branching abilities, we observed subtle changes when targeting the Hedgehog and Notch pathways. For example, administration of the GLI1/2 antagonist GANT61 led to cytotoxic effects, especially in the mesenchymal subtype, while the further upstream-acting Smoothened inhibitor SANT-1 did not affect the branching abilities of either subtype. Notch inhibition using the gamma secretase inhibitor, DAPT (10 µM), reduced the size for both organoid subtypes and affected the number of sub-branches of the epithelial subtype (Fig. 2a). The most pronounced effect was observed by manipulating TGFβ signalling. TGFβ is a crucial morphogen in pancreatic embryonic development as well as carcinogenesis^21^, particularly serving as a master regulator of EMT^22^. We have previously shown that EMT and mesenchymal-to-epithelial transition (MET) are dynamically regulated during branching morphogenesis of PDAC organoids^14^ and on the basis of the transcriptional profiling of matched 2D monolayers and corresponding 3D branched organoids, we identified TGFβ signalling, along with other EMT mediators such as Ski and Junb, to be significantly upregulated in the 3D organoids derived from both the epithelial and the mesenchymal clusters (Fig. 2b,c).

To assess the functional role of TGFβ-1 ligand in organoid morphogenesis, organoids were treated with TGFβ-1, the EMT-inducer cocktail STEMX^23^, or with a TGFβ-RI inhibitor A83-01. Organoids exposed to TGFβ-1 or STEMX from Day 0 of culture demonstrated growth arrest (size reduction) and a scattered phenotype for the epithelial organoids with reduced cell–cell contacts compared with control organoids. When TGFβ-1 was administered on Day 7, organoids displayed contractile and invasive phenotypes leading to extreme shrinkage of collagen gels, making organoid assessment by brightfield microscopy unfeasible. Therefore, we performed a 1-time treatment from Days 11–13 (48 h), which resulted in the formation of invasive spiky branches, indicating the cells undergoing EMT (Fig. 2d, epithelial upper panel). Conversely, inhibition of TGFβ-RI by A83-01 blocked organoid branch formation in both epithelial and mesenchymal tumour organoids (Fig. 2d).

In summary, TGFβ signalling is crucial for PDAC branched-organoid morphogenesis, as TGFβ inhibition abrogates branching completely. At the same time, the activation of TGFβ signalling at different timepoints of organoid development indicates a delicately orchestrated pathway, as stimulation at the beginning of organoid formation reduces branch number and thickness, whereas stimulation at later developmental stages leads to contraction of organoids.

### Branching PDAC organoids retain phenotypic specifications after transient EMT induction

**Extended Data Fig. 2:**
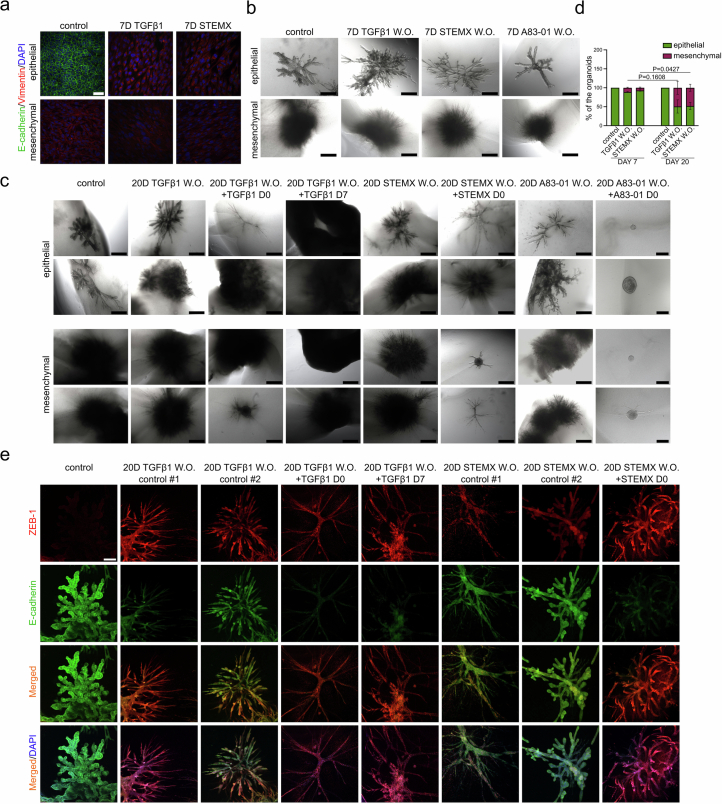
EMT induction via TGFβ signalling reveals heterogeneous responses. **a** Confocal IF imaging of E-cadherin (green), Vimentin (red) and DAPI (blue) for the epithelial (top) and mesenchymal (bottom) 2D cells (n = 3 mouse E and 3 mouse M lines) after 7 days EMT induction with 5 ng TGFβ1 or 1x STEMX, Scale bars= 50 μm. **b** Epithelial (top, from the mouse line ID: 9591) and mesenchymal (bottom, from the mouse line ID: 16992) organoid morphologies after treatment of the 2D cells for 7 days prior to seeding into organoid cultures with: 5 ng TGFβ1, 1x STEMX or 5 µM A83-01. Scale bars, 500 μm. **c** Epithelial and mesenchymal organoid morphologies after treatment of the 2D cells for 20 days prior to seeding into 3D-cultures with: 5 ng TGFβ1, 1x STEMX or 5 µM A83-01, additionally the treatment renewal with 1x STEMX, 5 µM A83-01 at day 0 and at specific days for TGFβ1: day 0, day 7, day 11 was included. All scale bars are 500 μm except from the 20 Days treated with A83-01 W.O. and then treated with additional A83-01 (most right column) where scale bars are 200 μm. **d** Quantification of epithelial and mesenchymal organoid morphologies in their W.O. phase from epithelial origin organoids (from the mouse line ID: 9591) after 7- or 20-days pre-treatment with 5 ng TGFβ1 or 1x STEMX (7 days treatment control n = 101, TGFβ1 W.O. n = 89 and STEMX W.O. n = 114 organoids from 3 individual experiments. 20 days treatment control n = 159, TGFβ1 W.O. n = 59 and STEMX W.O. n = 80 organoids). Unpaired two-tailed parametric t-test with Welch’s correction, two-tailed. **e** Confocal IF imaging of E-cadherin (green), Zeb1 (red) and DAPI (blue) for the epithelial organoids after 20 days of EMT induction with: 5 ng TGFβ1, 1x STEMX as control populations and with treatment renewal using 1x STEMX at day 0 and at specific days for TGFβ1: day 0 and day 7. The two main populations (without treatment renewal) are displayed, #1 the stable mesenchymal and #2 the reverting epithelial, Scale bars, 200 μm. Source data

Given the critical role of EMT in phenotypic diversity of organoids and TGFβ signalling in branching morphogenesis, we next tested the plasticity memory^24^ of tumour cells upon induction of EMT. First, we treated indicated PDAC cells in 2D monolayer cultures with TGFβ-1 and STEMX for 7 days, leading to EMT induction illustrated by increased Vimentin and loss of E-cadherin expression (Extended Data Fig. 2a). Subsequently, when de-differentiated cells were seeded in the branching organoid assay, the majority of organoids (88.7% after TGFβ and 92.8% after STEMX treatment) reverted to the phenotype of EMT state-of-origin (Extended Data Fig. 2b,d). Because previous studies suggest that a longer exposure to TGFβ can permanently transform epithelial PDAC cells to undergo EMT^25^ and that this is necessary for the maintenance of a mesenchymal phenotype, we chose to increase the exposure to 20 days. TGFβ-1 treatment for 20 days revealed the existence of 2 distinct epithelial subpopulations (Extended Data Fig. 2c): one that is plastic and reverts to the epithelial phenotype after TGFβ-1 withdrawal (accounting for 50.7% of the population after TGFβ-1 and 52% after STEMX treatment, top row of Extended Data Fig. 2c,d) and another that has limited plasticity memory and is unable to revert to the phenotype of the cell of origin (accounting for 49.3–48% of the respective TGFβ or STEMX treatment, second row of Extended Data Fig. 2c,d). Indeed, the organoids that are unable to revert maintained a nuclear Zeb1 expression accompanied by the loss of basolateral E-cadherin expression (20D TGFβ-1 W.O. control #1 and 20D STEMX W.O. control #1, Extended Data Fig. 2e), while the ‘plastic’ clones decreased/lost most of the Zeb1 nuclear localization and re-expressed E-cadherin (20D TGFβ-1 W.O. control #2 and 20D STEMX W.O. control #2, Extended Data Fig. 2e).

Taken together, these results demonstrate the impact that a master regulator of EMT, such as TGFβ signalling, can have on the generation of heterogeneous populations and that by chemically perturbating the signalling cascade, we can reveal plastic memory responses or stable transformations.

### Generation of a PDAC organoid phenotypic landscape

**Fig. 3:**
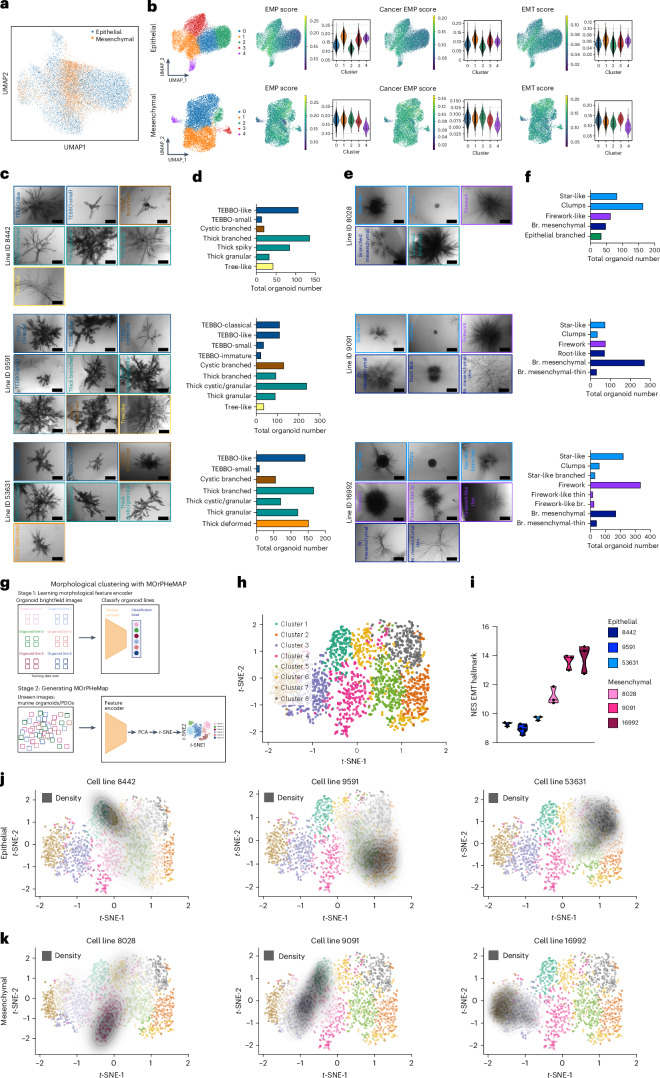
Intra-cell-line heterogeneity drives organoid phenotype diversity. **a**, UMAP plots from single-cell RNA sequencing of epithelial and mesenchymal 2D bulk populations. **b**, UMAP plots from single-cell RNA sequencing of epithelial 2D bulk populations (cell line ID: 9591, *n* = 16,747 cells) and mesenchymal 2D bulk populations (cell line ID: 16992, *n* = 9,190 cells). Conserved EMP, Cancer EMP according to the signature gene sets^26^ and EMT hallmark scores are presented in violin plots, the *y*-axis represents the AUCell scores for the specific pathways. **c**, Major morphologies found in epithelial organoid lines (*n* = 3 mouse lines, IDs: 8442, 9591, 53631). Colour coding implies the hierarchical relation of the super families. Scale bars, 500 µm. **d**, Manual clustering of the total number of organoids (line ID: 8442 *n* = 438, ID: 9591 *n* = 864, ID: 53631 *n* = 718 organoids). **e**, Major morphologies found in mesenchymal organoid lines (*n* = 3 mouse lines, ID: 8028, 9091, 16992). Colour coding implies the hierarchical relation of the super families. Scale bars, 500 µm. **f**, Manual clustering of the total number of organoids (line ID: 8028 *n* = 392, ID: 9091 *n* = 562 and ID: 16992 *n* = 900 organoids). **g**, Schematic representation of the workflow to develop MOrPHeMap; image created with BioRender.com. **h**, *K*-means clustering of the image-derived features of unseen data set of *n* = 1,579 organoids (from 6 mouse lines, 3-E IDs: 8442, 9591, 53631 and 3-M IDs: 8028, 9091, 16992) revealed 8 distinct morphological clusters. **i**, NES of the EMT hallmark from the 3 epithelial and 3 mesenchymal mouse lines grown in 3D collagen gels. **j**,**k**, Individual cell-line morphological heterogeneity as visualized by density overlays superimposed on the imaged-derived clusters. The overlays indicate which cell lines correspond to which cluster. **j**, *t*-SNE plots of the organoids clustering from the epithelial lines (IDs: 8442, 9591, 53631). **k**, *t*-SNE plots of the organoids clustering from the mesenchymal lines (IDs: 8028, 9091, 16992). Br. mesenchymal, branched mesenchymal; Br. mes.-thin, branched mesenchymal thin. Source data

**Extended Data Fig. 3:**
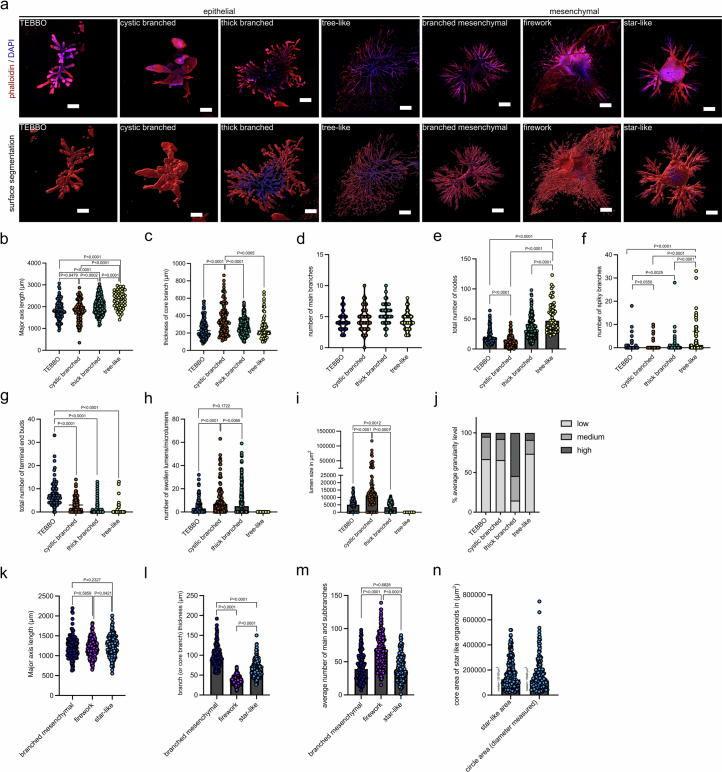
Structural characterization of distinct organoid phenotypes from epithelial and mesenchymal PDAC subtypes. **a** Phalloidin (red)/DAPI (blue) IF staining (top) of the distinct epithelial and mesenchymal organoid phenotypes. Surface segmentation of the above images with Imaris Scale bars= 300 μm for TEBBO, cystic branched, 500 μm for thick branched and tree-like, 400 μm for the branched mesenchymal and 200 μm for the firework and star-like organoids. **b-j** Morphological parameters analysis for epithelial (from the mouse line ID: 9591) organoid phenotypes: TEBBO (n = 106 organoids), cystic branched (n = 105 organoids), thick branched (n = 267 organoids) and tree-like (n = 57 organoids). **b** Major axis length of epithelial organoids in μm. **c** Thickness of core branches in μm. **d** Violin plot of average number of main branches. **e** Total number of nodes. **f** Average number of spiky branches. **g** Average number of terminal end buds. **h** Number of swollen lumens/microlumens **i** Lumen size of the epithelial organoid phenotypes measured in μm^2^. **b-c** and **e-i** Graphs represent median with interquartile range. Unpaired two-tailed non parametric t-test, Mann-Whitney test. **j** % average level of granular appearance of organoids (low, medium, high). **k-n** Morphological parameters analysis for mesenchymal (from the mouse line ID: 16992) organoid phenotypes: branched mesenchymal (n = 158 organoids), firework (n = 261 organoids) and star-like (n = 206 organoids). **k** Major axis length of mesenchymal organoids in μm. **l** Branch (or core branch) thickness measured in μm. **m** Average number of total branches (main and subbranches). **k-m** Graphs represent median with interquartile range. Unpaired two-tailed non parametric t-test, Mann-Whitney test. **n** Comparison of the core area of star-like organoids to the theoretical perfect circular area by measuring the diameter (radius=diameter/2) of the organoids and then calculating the area (A), A= π*r^2^. Graph represents median with interquartile range. Source data

To determine the pre-existing heterogeneity of the parental PDAC cells cultured in 2D, we performed single cell RNA-sequencing (scRNA-seq) of primary epithelial and mesenchymal PDAC lines displaying a continuum of cell states (Fig. 3a). When the transcriptional subtypes were analysed separately, 5 main clusters (clusters 0–4) were identified for both the epithelial and mesenchymal PDAC subtypes. Next, we analysed the epithelial-mesenchymal plasticity (EMP)/EMT score distribution^26^ of distinct clusters (Fig. 3b). Interestingly, we observed the presence of heterogeneous EMP/EMT scores between these clusters. It has been shown previously that tumour heterogeneity is driven by pre-existing EMT transition states in other cancer entities^27^ and that in PDAC, multiple phenotypes can co-exist across the EMT spectrum^6,28,29^. Given the observed phenotypic diversity and heterogeneity of organoids derived from distinct transcriptional PDAC subtypes, and the existence of predetermined cell states within the 2D lines, we next aimed to determine whether organoids derived from the same transcriptional subtype display an inherent phenotypic heterogeneity in our assay. First, we visually observed the formation of several distinct morphological subclusters (Fig. 3c,e). In detail, we analysed organoid morphologies from three epithelial PDAC lines (*n* = 2,020 organoids derived from ID: 8442, ID: 9591 and ID: 53631, Fig. 3d) and three mesenchymal PDAC lines (*n* = 1,854 organoids from ID: 8028, ID: 9091 and ID: 16992, Fig. 3f). Among both PDAC subtypes, we found conserved organoid phenotypic families. For the epithelial subtype, we defined 4 main morphologic categories/families, namely, the terminal end bud branching organoid (TEBBO), cystic branched, thick branched and tree-like family, with every category representing a different proportion of the entire organoid population (Extended Data Fig. 3a, left panel). In general, branching epithelial organoids consist of a main branch with multiple sub-branches. To determine the unique structural features of each epithelial category, we quantified the organoid size as major axis length, thickness of the core branch and number of main branches, total number of nodes (branch points), the presence or absence of invasive protrusions (spikey branches), end tubular structures, lumen formation (number of swollen lumens/micro-lumens and total lumen area) and granularity (Extended Data Fig. 3b–j). Indeed, key characteristics exist among the different organoid categories. For instance, the TEBBO family has the most terminal end buds (Extended Data Fig. 3g), the cystic branched family has the most micro-lumens and the largest lumen area (Extended Data Fig. 3h,i), the thick branched family displays the highest granularity levels (Extended Data Fig. 3j) and the tree-like family displays the highest number of nodes and the most invasive branches (Extended Data Fig. 3e,f).

Mesenchymal organoids derived from the C1 cluster exhibited reduced phenotypic diversity with 3 main morphological categories: the branched-mesenchymal, the firework and the star-like phenotypes (Extended Data Fig. 3a, right panel). In the mesenchymal organoids, we quantified the organoid size as major axis length, branch (or core branch) thickness and total number of branches (Extended Data Fig. 3k–m). Unique characteristics include the presence of a thick core branch in the branched-mesenchymal category (Extended Data Fig. 3l), the firework organoids having the highest number of branches (Extended Data Fig. 3m) and, notably, the star-like organoids forming a circular core representing an almost perfect circle (Extended Data Fig. 3n).

In summary, using our branching organoid assay, we are able to capture pre-existing tumour cell heterogeneity, which is correlated with EMT transition states. Our culture conditions foster a remarkable phenotypic heterogeneity, ranging from well-differentiated structures with thick branches and terminal end buds (TEBBOs), reminiscent of the pancreatic ductal tree, to highly invasive organoids (firework/star-like) with hundreds of invasive branches. Culturing branching organoids at clonal densities results in reproducible phenotypes with unique structural features underscoring the functional heterogeneity between and, importantly, within distinct PDAC subtypes.

### Mapping phenotypic heterogeneity of PDAC organoids across different transcriptional subtypes and different genotypes

**Extended Data Fig. 4:**
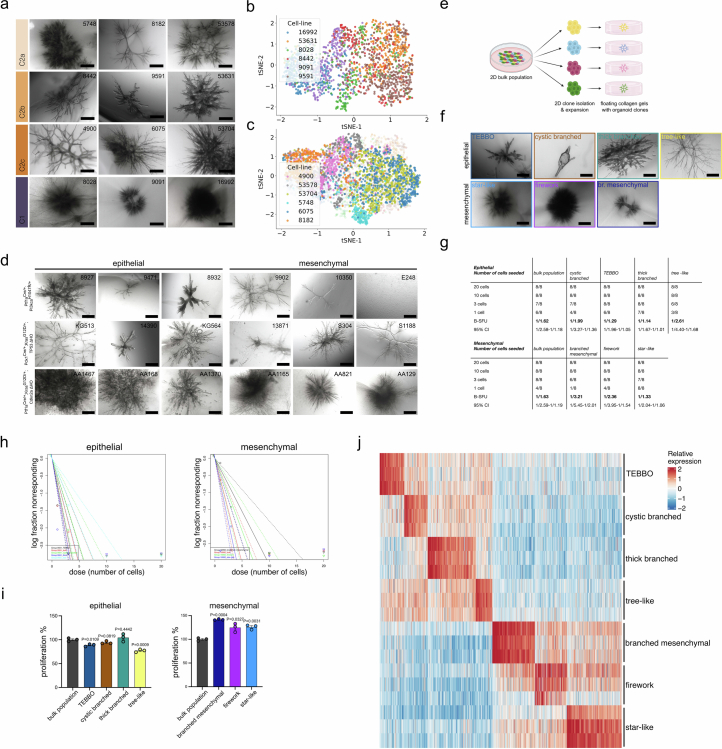
Deciphering inter-cell and intra-cell organoid heterogeneity. **a** Characteristic organoid morphologies derived from four different transcriptional clusters as previously described^15^ (C2a, C2b, C2c, C1). Scale bars= 500 μm. **b** t-SNE plot clustering showing the individual organoid lines from Fig. 3h. **c** t-SNE plot clustering for the C2a and C2c organoids (n = 2015 organoids) superimposed on the Extended Data Fig. 4b. **d** Organoid phenotypes (n = 588 organoids) from epithelial and mesenchymal type of organoids derived from various mouse models (with different driver mutations): Ptf1a^Cre/+^; Pi3kca^+H1047R/+^ (n = 6 mouse lines, note that the line E248 is not forming 3D organoids), Pdx1^Cre/+^; Kras^G12D/+^; TP53 ΔHO (n = 6 mouse lines), Ptf1a^Cre/+^; Kras^G12D/+^; Cdkn2a ΔHO (n = 6 mouse lines). Scale bars= 500 μm. **e** Schematic representation of the morphological clone isolation and expansion. Image created with BioRender.com. **f** Major 3D morphological families that were used for further characterisation. Scale bars= 500 μm. **g** Extreme Limiting Dilution Analysis (ELDA) of epithelial and mesenchymal organoid phenotypes. **h** Log plot of nonresponding wells vs cell dosage for the epithelial (left) and mesenchymal (right) 3D organoid phenotypes. **i** Proliferation rate as % compared to the bulk population for the epithelial (from the mouse line ID: 9591 from 3 individual experiments) and mesenchymal (from the mouse line ID: 16992 from 3 individual experiments) 2D clones. Unpaired two-tailed parametric t-test with Welch’s correction, two-tailed. Both graphs represent mean±sem. **j** Heatmap of signature genes from E and M organoid phenotypes. Source data

After having identified the existence of multiple distinct organoid phenotypes derived from a panel of PDAC cell lines across different tumour transcriptional subtypes and manually categorizing them on the basis of morphologies, we developed an unbiased machine learning approach to capture and quantify phenotypic heterogeneity of single-cell-derived PDAC organoids. Specifically, we applied deep convolutional neural networks and topological machine learning techniques to generate a statistical representation of PDAC organoid phenotype heterogeneity, which we term Morphologic Organoid Phenotypic Heterogeneity Mapping (MOrPHeMap). MOrPHeMap was generated using a fixed (neural-network-based) feature extractor, which was initially trained using 4,113 individual organoid images to classify the organoid images into their respective cell lines (Fig. 3g). This network was then used to extract semantically relevant features from an unseen set of 1,579 organoid images (Extended Data Fig. 4b), which were then visualized using *t*-distributed stochastic neighbour embedding (*t*-SNE) to obtain a two-dimensional, spatial representation of the recognized clusters (Fig. 3h) and their position along an EMT spectrum (Fig. 3i). Interestingly, the resulting 8 major clusters closely matched our manual analysis, as seen in Fig. 3c,e. Next, when superimposing the cell line identity to MOrPHeMap, the phenotypic diversity of organoids derived from individual tumours becomes evident. For example, the epithelial PDAC lines (ID: 9591, 53631) co-localize (right) in the *t*-SNE plot, whereas the mesenchymal PDAC lines (ID: 9091, 16992) cluster to the opposite (left) side, both displaying multiple morphological clusters (Fig. 3j,k). Of note, ID: 8028, which is the mesenchymal line with the lowest EMT score by bulk RNA sequencing (Fig. 3i), generates a small population of epithelial (8.67%) organoid morphologies in 3D (Fig. 3e,f) and, indeed, this phenotypic diversity is also captured by larger numbers of morphological clusters in MOrPHeMap (Fig. 3k). Next, we embedded single cells from an expanded panel of *Kras*^*G12D*^-driven PDAC lines (*n* = 6 mouse lines) as described previously^15^, including not only the abovementioned transcriptional extremes of mesenchymal and epithelial PDAC cells, but also a continuum of the epithelial subclusters, termed cluster 2 a–c (C2a–c) (Extended Data Fig. 4a). We applied the neural network to this expanded repertoire of PDAC organoids comprising an unseen set of 2,015 organoids (Extended Data Fig. 4a,c). The majority of the C2c-derived organoids (line IDs: 53704 and 6075) cluster together on the right side of the *t*-SNE and the C2a-derived organoids (line IDs: 8182 and 53578) towards the left side of the *t*-SNE (Extended Data Fig. 4c). Interestingly, the line ID: 4900, although originally characterized as C2c line (transcriptionally), morphologically clusters together with the C2a PDAC organoids, while the line ID: 5748 (from the C2a transcriptional cluster) clusters with the C2c organoids, indicating that MOrPHeMap is able to resolve morphological differences within the transcriptional PDAC subtypes.

We have demonstrated so far that pre-existing transcriptional heterogeneity, in particular EMT states, contributes to phenotypic organoid diversity. To determine whether different PDAC driver mutations impact the morphogenesis of distinct organoid phenotypes^30^, we analysed, in addition to PDAC cell lines generated from KC mouse models, a panel of primary PDAC cells (*n* = 18 PDAC mouse lines) derived from tumours harbouring *Kras*^*G12D*^ and *Cdkn2a* (*n* = 6 lines) or *Trp53* (*n* = 6 lines) deletions as well as tumours with a *Pik3ca*^*H1047R*^ activating mutation^31^ (*n* = 6 lines). Interestingly, when generating single-cell-derived organoids from primary cell lines from these PDAC mouse models, we observe a substantially similar spectrum of phenotypes as with KC-derived organoids, indicating that the phenotypic subtypes are rather determined by the transcriptional programmes than by the genetic driver mutations (Extended Data Fig. 4d).

Taken together, MOrPHeMap captures intertumoural and, most importantly, intratumoural organoid diversity. The profound phenotypic diversity of organoids derived from the same PDAC tumour cell lines suggests functional differences and could serve as a model system for intratumoural heterogeneity.

### Heterogeneity within PDAC organoids is orchestrated by distinct transcriptional programmes and characterized by functional diversity

Pre-existing heterogeneity defines clonal populations of cells. In addition, non-genetic plasticity enhances somatic evolution of cancer cells, thus promoting tumour progression independently of the genetic mutation^32^. Since we were able to demonstrate the existence of morphological heterogeneity within organoids derived from the same PDAC mouse lines, we next sought to identify transcriptional programmes defining these heterogeneous organoid morphologies.

**Fig. 4:**
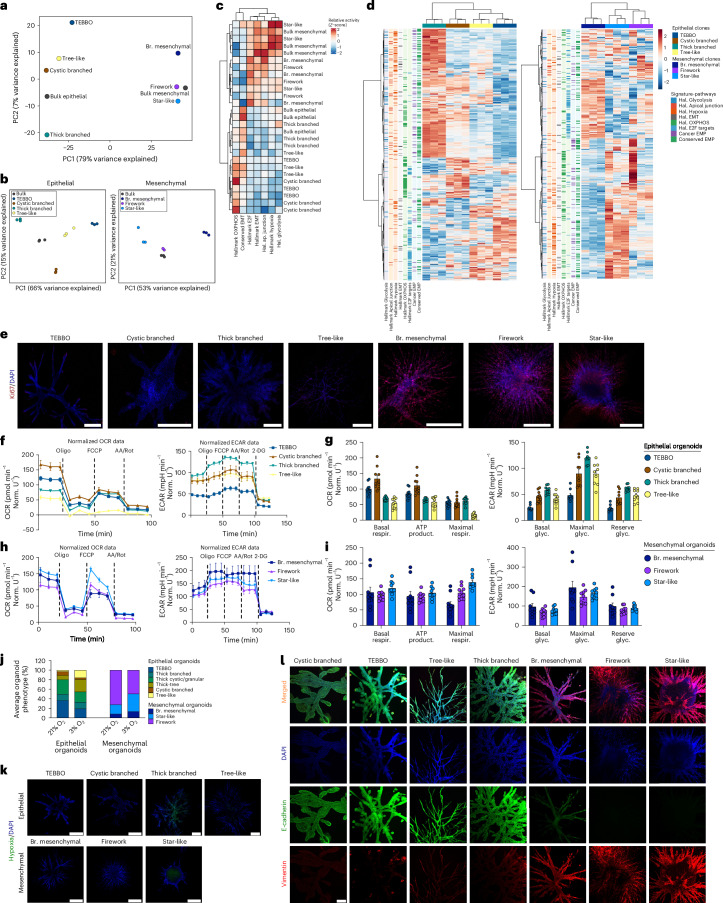
Molecular and functional characterization of distinct organoid morphologies. Transcriptomic and pathway analyses from individual organoid phenotypes isolated from the E-mouse line ID: 9591 and the M-mouse line ID: 16992. **a**, PCA of bulk RNA sequencing from different morphological epithelial and mesenchymal organoids. Each dot represents the mean of 3 independent experiments. **b**, Subtype-specific PCA of the bulk RNA sequencing from different morphological epithelial (left) and mesenchymal organoids (right). **c**, Heat map score activity of epithelial and mesenchymal organoids. **d**, Heat map of the hallmarks: Glycolysis, Apical Junction, Hypoxia, EMT, Oxidative phosphorylation, E2F targets, EMP and Cancer EMP^26^ characterizing the individual clonal epithelial (left) and mesenchymal (right) organoids. **e**, Immunofluorescence staining for the proliferation marker Ki67 in epithelial and mesenchymal organoids. Scale bars, 500 µm. **f**, Seahorse OCR and ECAR measurements in distinct epithelial organoid phenotypes. **g**, Quantification of basal respiration, ATP production and maximal respiration in the OCR (left), and basal, maximal and reserved glycolysis in the ECAR (right) normalized data for distinct epithelial organoid phenotypes. **h**, Seahorse OCR (left) and ECAR (right) measurements in distinct mesenchymal organoid phenotypes. **i**, Quantification of basal respiration, ATP production and maximal respiration in the OCR (left), and basal, maximal and reserved glycolysis in the ECAR (right) normalized data for distinct mesenchymal organoid phenotypes. **f**–**i**, *n* = 10 technical replicates for the OCR and *n* = 9 technical replicates for the ECAR measurements for both epithelial and mesenchymal organoid phenotypes. Graph represents mean ± s.e.m. **j**, Manual phenotype analysis of organoids in normal and hypoxic (3% O_2_) conditions for epithelial and mesenchymal organoids. *n* = 189 epithelial (3 individual experiments) and 235 mesenchymal (3 individual experiments) organoids. Bar plot represents the average number of organoid phenotypes (%). **k**, In vitro hypoxia confocal imaging. Staining was performed with the fluorescent Image-iT green hypoxia reagent (Thermo Fisher) and DAPI, *n* = 2 individual experiments. Scale bars, 500 µm. **l**, Confocal IF imaging of E-cadherin (green), Vimentin (red) and DAPI (blue) for the 4 major epithelial morphological clones: cystic branched, TEBBO, tree-like and thick branched (from left to right), and the 3 major mesenchymal morphological clones: branched mesenchymal, firework and star-like (from left to right). Scale bars, 200 µm. Br. mesenchymal, branched mesenchymal; respir., respiration; glyc., glocolysis; product., production. Source data

To this end, we isolated clonal organoid phenotypes from all major morphological categories (4 epithelial and 3 mesenchymal) (Extended Data Fig. 4e,f) and performed transcriptomic analysis. Principal component analysis (PCA) of clonal organoid phenotypes compared to parental bulk tumour organoids revealed a pronounced difference in global gene expression depending on individual phenotypic clones and subtype (Fig. 4a,b). Differences in key cellular processes include: (1) proliferation (via E2F targets upregulated in the branched-mesenchymal and tree-like phenotypes), (2) metabolism (glycolysis upregulated in the thick branched and star-like phenotypes and oxidative phosphorylation upregulated in cystic branched and star-like phenotypes), (3) hypoxia (upregulated in thick branched and star-like organoids) and (4) apical junctions (upregulated in the thick branched organoids) (Fig. 4c,d).

Next, we functionally validated these pathways (1–4). We observe a gradient of proliferation from low proliferating organoid phenotypes (cystic branched, thick branched, TEBBO) to intermediate (tree-like) and the highly proliferating mesenchymal organoids (branched mesenchymal, firework, star-like) (Fig. 4e). Metabolically, epithelial organoid phenotypes exhibit prominent differences, with the cystic branched organoids having the highest basal respiration and ATP production, while the thick branched organoids rely more on total glycolysis (basal/maximal and reserve) for their energy production (Fig. 4f,g). Mesenchymal organoids express marginal differences, with the star-like organoids showing an enrichment of their oxygen consumption rate (OCR; especially their maximal respiration) and the branched-mesenchymal organoids producing more ATP via glycolysis (basal, maximal and reserve glycolysis) (Fig. 4h,i). Next, we tested whether hypoxic conditions (3% O_2_) would favour the generation of specific phenotypes in the bulk populations. Indeed, the most enriched phenotype for the hypoxia signature within the mesenchymal PDAC subtype, the star-like organoid, was enriched to 37% of the total population under hypoxia conditions compared with 19.4% under normoxic conditions (21% O_2_) (Fig. 4j). To further validate whether hypoxia was induced by the phenotypes and not prerequired to form a specific phenotype, we visualized low oxygen environments in the organoids using hypoxia stain (Image-iT green hypoxia reagent), finding the thick branched (epithelial) and the star-like (mesenchymal) phenotypes to be the most hypoxic (Fig. 4k). Epithelial-to-mesenchymal transition and EMT plasticity are milestones of tumour progression and treatment resistance^26,33^, and their different expression/commitment levels contribute to the formation of heterogeneous organoid morphologies. We stained for the epithelial marker E-cadherin and the mesenchymal marker Vimentin. We observed a gradual decrease in E-cadherin expression from the very epithelial cystic branched organoids, via TEBBOs to thick branched and eventually the highly mesenchymal star-like organoid phenotypes, whereas Vimentin expression shows an inverse pattern (Fig. 4l).

In general, distinct EMT differentiation states are frequently associated with unique self-renewal capabilities and stemness^34^. In line with this, in vitro, the morphological clones display different capacities to form multicellular structures (branching-structure formation units (B-SFUs)) in 3D conditions. Thick branched organoids display the highest (B-SFU 1/1.67–1/1.01 95% CI) and tree-like organoids the lowest (B-SFU 1/4.4–1/1.68 95% CI) organoid formation capacity within the epithelial subcluster. For the mesenchymal subcluster, the star-like organoids are the most potent (B-SFU 1/2.04–1/1.06 95% CI) and the branched mesenchymal organoids represent the least potent (B-SFU 1/5.45–1/2.01 95% CI) in forming organoids (Extended Data Fig. 4g,h) independent of their proliferation capacities (Extended Data Fig. 4i).

Taken together, this indicates that phenotypic heterogeneity of PDAC organoids is attentively directed by the expression of distinct transcriptional programmes, and this translates to distinct basal cellular functions.

### Distinct organoid phenotypes correlate with different tumour cell states and properties

**Fig. 5:**
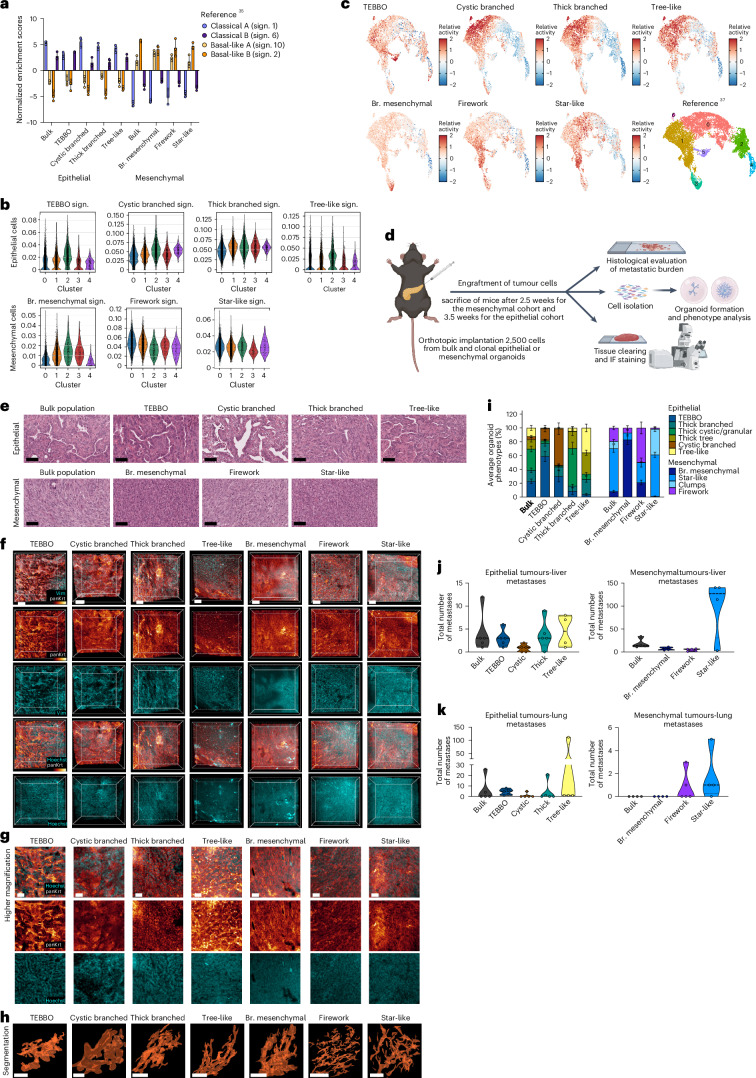
Organoids phenotypes represent distinct tumour cell states with unique in vivo biological functions. **a**, Scoring of the individual organoid phenotypes isolated from the E-mouse line ID: 9591 and the M-mouse line ID: 16992 for PDAC subtype-specific signatures^35^. Graph represents mean ± s.e.m., each dot represents an independent experiment. **b**, Violin plots of scRNA-seq from the parental epithelial and mesenchymal cells scored for the organoid phenotype signatures. The *y*-axis represents the AUCell scores for the specific signatures. **c**, UMAP scoring the individual organoid phenotype signatures to a human PDAC data set^37^. **d**, Schematic representation of the in vivo orthotopic transplantation and the subsequent analysis, including histopathological analysis, whole-tissue clearing and IF staining, and organoid line isolation/characterization; image created with BioRender.com. **e**, H&E staining of orthotopically transplanted organoids. Scale bars, 60 µm. **f**–**h**, 3D in vivo growth patterns of PDAC organoids. **f**, 3D views of PDAC organoid grafts stained for pan-Keratin and Vimentin. All scale bars, 100 μm. **g**, High magnifications of pan-Keratin from the PDAC grafts in **f**, demonstrating different growth patterns of the various organoid lines. All scale bars, 20 μm. **h**, 3D segmentations of coherent tumour cell strands from different organoid grafts. All scale bars, 30 μm. **i**, Manual phenotype analysis of organoids post implantation (*n* = 41 lines, *n* = 1,171 organoids from the epithelial lines and *n* = 633 organoids from the mesenchymal lines). Bar plot represents the mean ± s.e.m. of the average number of organoid phenotypes (%). **j**, Truncated violin plots of the total number of metastatic nodules in the liver from epithelial (left) and mesenchymal (right) transplanted organoid phenotypes. **k**, Truncated violin plots of the total number of metastatic nodules in the lung from epithelial (left) and mesenchymal (right) transplanted organoid phenotypes. Br. mesenchymal, branched mesenchymal; sign., signature. Source data

Human PDAC can be stratified into 2 major molecular subtypes with distinct characteristics and survival rates: the classical and basal-like PDAC^3^, and subsequent studies suggest that these two subtypes can be further divided into at least two subclusters: A and B (ref. ^35^). When we correlate the expression profiles of organoid phenotypes with these molecular subtypes, we noted a clear enrichment of signature 1 (classical-A) for the cystic branched organoids and signature 6 (classical-B) for the TEBBO organoids. In the mesenchymal subtype, the branched-mesenchymal organoids were enriched for signature 2 (basal-like A), while the star-like organoids were more enriched for signature 10 (basal-like B), suggesting a distinct biology of the different organoid phenotypes (Fig. 5a).

In PDAC progression, multiple tumour cell states can co-exist, and the emergence of these states represents key evolutionary events^36^. We next correlated the intra-cell-line transcriptional subclusters of epithelial and mesenchymal tumour cells with the signatures (Extended Data Fig. 4j) of the organoid phenotypes. In the epithelial cells, TEBBO signatures were enriched in cluster 2, while the cystic branched were enriched in clusters 4 and 2. In the mesenchymal cells, branched mesenchymal signatures were enriched in clusters 2 and 3, while firework signatures were enriched in clusters 0 and 1, and star-like signatures in cell cluster 4 (Fig. 5b and see also previous scRNA-seq in Fig. 3a,b). This indicates that specific organoid phenotype signatures were already enriched in defined subpopulations of the parental tumour lines.

To further define whether distinct organoid signatures are biologically relevant and occur in vivo as different tumour cell states in human disease, we evaluated the expression of organoid signatures in malignant cells in human PDAC scRNA-seq data^37^. Interestingly, several of the organoid phenotype signatures reflected distinct malignant states observed in human PDAC (Fig. 5c). For example, genes associated with the TEBBO phenotype were maximally expressed in a rare population of cells (Cluster 5). Firework and star-like genes were activated in a population distinct from those expressing high levels of epithelial (cystic branched, thick branched and tree-like) signatures, consistent with intratumoural EMP (Fig. 3b).

**Extended Data Fig. 5:**
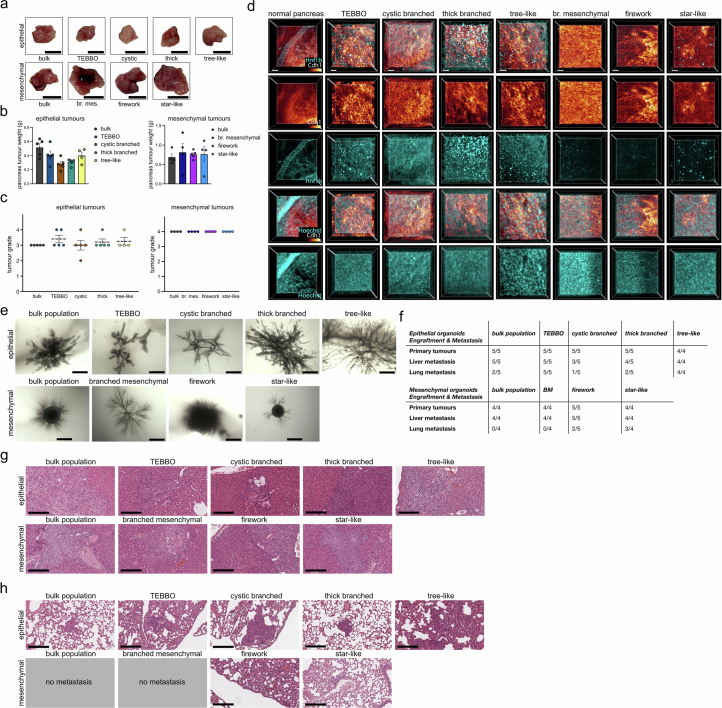
Orthotopic transplantation of organoid phenotypes. **a** Images of epithelial and mesenchymal tumours after transplantation of distinct organoid phenotypes. Scale bars= 1cm. **b-c** n = 41 mice, for the epithelial transplanted organoids n = 5 mice/phenotype (tree-like n = 4 mice) and for the mesenchymal transplanted organoids n = 4 mice/phenotype (firework n = 5 mice). **b** Pancreas tumour weight (g) for epithelial and mesenchymal organoid phenotypes derived tumours. Graphs represent mean±sem. **c** Tumour grading for epithelial and mesenchymal organoid phenotypes derived tumours. Graphs represent mean±sem. **d** *In vivo* marker expression of different transplanted organoid phenotypes. 3D views of *in vivo* PDAC organoid grafts stained for E-cadherin and Hnf1β. Note absent nuclear Hnf1β staining in mesenchymal organoid lines. All scale bars= 20 μm. **e** Characteristic organoid morphologies from cell lines isolated post-implantation (epithelial on top, mesenchymal bottom). Scale bars= 500 μm. **f** Summary table of the tumour engraftment and liver/lung metastatic colonization efficacy from epithelial and mesenchymal transplanted organoids in mice. BM: branched mesenchymal. **g** HEs of liver metastasis from epithelial (top) and mesenchymal (bottom) organoid phenotypes transplanted in mice. Scale bars= 200 μm. **h** HEs of lung metastasis from epithelial (top) and mesenchymal (bottom) organoid phenotypes transplanted in mice. Scale bars= 200 μm. Source data

To address this observation functionally, we determined the ability of tumour cells arising from distinct organoid phenotypes to establish tumours in the pancreas and metastasize in vivo. In addition, we investigated whether cells from distinct organoid phenotypes retain their morphological properties in vivo and, to assess how stable these tumour states are, we analysed organoid phenotypes after transplantation in vitro. In detail, we orthotopically implanted tumour cells from the distinct organoid phenotypes and the bulk organoid populations as controls (*n* = 9 phenotypes 5-E and 4-M) into immunocompetent mice of a syngeneic background (Fig. 5d). All transplanted mice (*n* = 41) developed tumours (Extended Data Fig. 5a) with no significant differences in the tumour weight (Extended Data Fig. 5b). As expected, analysis of the tissue architecture by H&E staining (Fig. 5e) revealed a clear separation between tumours derived from epithelial and mesenchymal transplanted organoids. In terms of tumour grade, mice transplanted with epithelial organoids developed more differentiated tumours (average histological grading 3) compared with those transplanted with mesenchymal organoids (average histological grading 4) (Extended Data Fig. 5c).

To further correlate the tumour architecture in vivo with the corresponding organoid phenotypes, we performed whole-tissue clearing and immunofluorescence (IF) staining^38^. Comparing epithelial (E) to mesenchymal (M) engrafted tumours, we observe a clear decrease in the expression and membranous localization of E-cadherin accompanied by the loss of the ductal lineage-marker HNF1B^4,38^ (Extended Data Fig. 5d). Of note, in H&E stains of tumours derived from distinct organoid phenotypes, we observed unique morphologies of tubular (ductal) structures within the tumour tissues depending on the transplanted organoid phenotype (Fig. 5e). The similarities in morphology between organoid phenotypes and corresponding tumours become even more prominent when analysing the tumour architecture of cleared whole tissues by IF staining for pan-Keratin and Vimentin (Fig. 5f,g), followed by segmentation of coherent tumour cell strands based on the pan-Keratin expression (Fig. 5g,h). In detail, we observed highly conserved structures mimicking the in vitro parental organoids (see also Extended Data Fig. 3a). Specifically, tumours derived from the epithelial TEBBO family and the cystic branched family share multiple key features with their parental organoids including thick branches, swollen lumens (prominent especially in the cystic branched tumours) and end tubular structures (Fig. 5h). In contrast, tumours derived from the thick branched family display decreased epithelial organization and lack end tubular structures (see also Extended Data Fig. 3a,g). The tree-like family forms complex structures in vivo, with multiple thin branches and sub-branches (see also Extended Data Fig. 3a,e). Similarly, mesenchymal organoid phenotypes share common morphological features with in vivo tumours. The branched mesenchymal family generates dense tumour cores with invading branches to the surrounding tissue, the firework family forms tumours with numerous thin branches and the star-like family gives rise to thick invading branches (Fig. 5g,h and see also Extended Data Fig. 3a,l,m).

To test the plasticity and EMT memory of organoid phenotypes after transplantation, we established 41 new organoid lines from the orthotopic tumours (post implantation) and analysed the morphological phenotypes in vitro (Extended Data Fig. 5e). Importantly, organoids from tumours from bulk populations without enrichment for any specific phenotype display the original morphological organoid heterogeneity, giving rise to the entire organoid phenotypic spectrum (both for E and M subtypes). Tumours from distinct organoid phenotypes generate organoids highly enriched for the phenotype of the cell (organoid) of origin for both E and M subtypes, showing a high degree of stability and retentive EMT memory of individual organoid phenotypes (Fig. 5i). Interestingly, tumours derived from the tree-like family gave rise mostly to tree-like organoids (35.9%); however, the second most common organoid phenotype represents a thicker type of tree-like (thick-tree) organoid (30.8%), reminiscent of the corresponding in vivo phenotype as illustrated by segmentation images of pan-Keratin (see also Fig. 5h).

Although distinct organoid phenotypes share tumour initiating capacities, with all transplanted mice developing tumours and displaying similar tumour weight, systematic histological analysis of common sites of metastatic colonization, such as the liver and lungs, revealed differential metastatic capacities for tumours derived from distinct organoid phenotypes (Extended Data Fig. 5f). For instance, within the epithelial organoid family the tree-like organoids show the highest metastatic capacity (liver mean = 4.5 nodules, lung mean = 28.75 nodules), whereas the cystic branched organoids harbour the lowest metastatic potential (liver mean = 0.8 nodules, lung mean = 1 nodule). Notably, thick branched and tree-like organoids represent more hybrid EMT phenotypes. Similarly, within the mesenchymal family of organoids, the star-like organoid phenotype gives rise to the most metastatic nodules (liver mean = 99.25, lung mean = 1.75) (Fig. 5j,k and Extended Data Fig. 5g,h).

In summary, we demonstrate that both epithelial and mesenchymal PDAC subtypes and their individual organoid phenotypes represent distinct cellular states, detectable also in human PDAC, and recapitulate their individual morphologies to a remarkable degree in vivo. Moreover, organoids isolated post implantation generate morphologically stable structures reminiscent of the organoid of origin. The metastatic capacity between organoid phenotypes varies substantially while having the same tumour initiating capacities. Importantly, higher EMT scored epithelial organoid phenotypes (thick branched, tree-like) and the highest EMT scored mesenchymal organoid phenotype (star-like) developed the most metastases, highlighting the impact of intratumoural heterogeneity with distinct cell states on metastatic dissemination.

### Phenotypic subclones harbour distinct therapeutic vulnerabilities to chemotherapy and radiation therapy

**Fig. 6:**
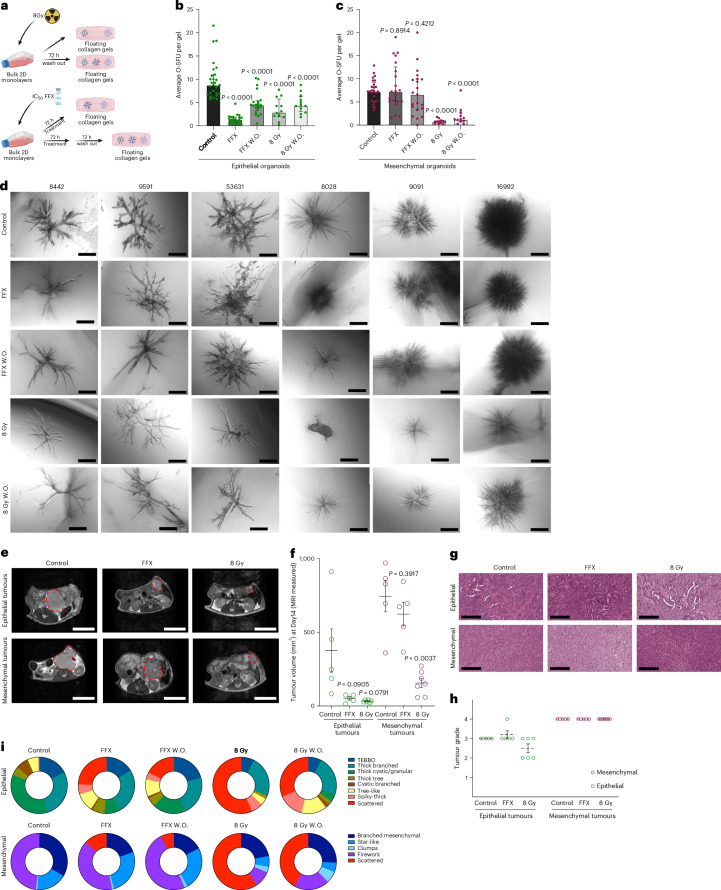
Defining PDAC subtype and organoid phenotype-specific vulnerabilities to radio- and chemotherapy. **a**, Schematic representation of the in vitro workflow for the different treatment approaches with either FOLFIRINOX (FFX) IC_50_ values or 8 Gy irradiation; image created with BioRender.com. **b**,**c**, Average O-SFU per gel for epithelial (**b**) and mesenchymal (**c**) type of organoids. All organoid numbers correspond to 3 E-lines (IDs: 8442, 9591, 53631) and 3 M-lines (IDs: 8028, 9091, 16992). For all E and M-lines control *n* = 9, FFX *n* = 7, FFX W.O. *n* = 7, 8 Gy *n* = 4 and 8 Gy W.O. *n* = 4 individual experiments (except for the M-line ID: 8028 where control *n* = 7 individual experiments). Graphs represent median with interquartile range. Unpaired two-tailed non-parametric *t*-test, Mann–Whitney test. **d**, Representative organoid morphologies of epithelial (from 3 mouse lines) and mesenchymal (from 3 mouse lines) organoids before (control *n* = 2,438 organoids), after treatment with FFX (*n* = 1,470 organoids), 8 Gy irradiation (*n* = 428 organoids) or their washout phases (FFX W.O. *n* = 1,594 and 8 Gy W.O. *n* = 638 organoids). Scale bars, 500 µm. **e**, MRI images of the in vivo tumours at Day 14 after transplantation for epithelial (top) and mesenchymal (bottom) transplanted lines. Scale bars, 1 cm. **f**, Quantification of the tumour volume from the MRI measurements. Graph represents mean ± s.e.m, unpaired two-tailed parametric *t*-test with Welch’s correction, two-tailed. **g**, Representative H&E images of the transplanted tumours. Scale bars, 200 µm. **h**, Histological grading of the transplanted tumours (*n* = 33 mice); graph represents mean ± s.e.m. **i**, Manual phenotypic analysis of epithelial (*n* = 915 organoids, from the mouse line ID: 9591 from at least 3 individual experiments) and mesenchymal (*n* = 1,257 organoids, from the mouse line ID: 16992 from at least 3 individual experiments) organoids before, after treatment and in the respective washout phase. Pie charts represent the average number of organoid phenotypes (%). Source data

PDAC organoids have been shown to harbour intra-organoid heterogeneity on a transcriptional level^39^; however, the impact of this heterogeneity on treatment response has not been investigated in detail. Therefore, we next employed our model system to test whether phenotypic subclones harbour distinct therapeutic vulnerabilities. First, we exposed PDAC cells to either a standard-of-care polychemotherapy, FOLFIRINOX [(folate), fluorouracil, irinotecan hydrochloride, oxaliplatin], or irradiation (8 Gy). In detail, PDAC cells from epithelial and mesenchymal PDAC subtypes were pretreated with their respective half-maximal inhibitory concentration (IC_50_)values of FOLFIRINOX for 72 h in 2D and then seeded into floating collagen gels, termed FFX treated. To determine the recovery of certain phenotypes after treatment and investigate temporal effects on phenotypic diversity and plasticity in PDAC organoids, we included a ‘washout’ (FFX W.O.) group in which, after treatment with FOLFIRINOX for 72 h, media were changed to normal culture media conditions for 72 h before seeding the cells into gels. For the irradiation treatment, we followed a similar approach, where PDAC cells were irradiated with 8 Gy and then directly seeded into floating collagen gels, termed 8 Gy organoids, or PDAC cells were first irradiated with 8 Gy and then left under normal conditions to recover for 72 h before being seeded into gels, termed 8 Gy ‘washout’ organoids (8 Gy W.O.) (Fig. 6a). First, we quantified the organoid structure formation units (O-SFUs) of (1) control organoids (control), (2) organoids after exposure to FFX or irradiation (FFX/8 Gy) and (3) washout groups (FFX W.O./8 Gy W.O.) (Fig. 6b,c). For the PDAC subtypes, epithelial PDAC cells were strongly affected by FFX chemotherapy that significantly reduced their capacity to form organoids by 85.3%, while the mesenchymal PDAC organoids were largely unaffected. In contrast, irradiation (8 Gy) had a stronger impact on the mesenchymal PDAC cells, as the organoid formation capacity was reduced significantly by 89.6% compared with 64.3% for the epithelial PDAC organoids (Fig. 6b,c).

We next focused on changes in organoid morphology after treatment (FFX or 8 Gy) and their respective washout conditions. In line with the previous analysis, we observed that the morphology of mesenchymal organoids is mostly unaffected by FFX treatment, while epithelial organoids display size reductions and paucity of branches in the FFX-exposed group (Fig. 6d). In contrast, irradiation (8 Gy) resulted in a notable organoid structure size and branch number reduction for the mesenchymal organoids and recovery of organoid morphology after the washout phase. The epithelial organoids displayed smaller organoids with thinner and fewer branches compared with the control after irradiation, with the washout phase partially rescuing their thickness (Fig. 6d). These results indicate PDAC organoid subtype-specific response to standard-of-care therapy and rapid phenotype regeneration upon discontinued treatment.

Corroborating these results, important findings from the COMPASS trial indicate that the classical PDAC subtype corresponding to more differentiated (epithelial) tumours is more sensitive to FFX polychemotherapy compared with the basal-like, more mesenchymal subtype^40^. Therefore, and to further validate our in vitro findings in an in vivo setting, we pretreated cells with FFX or irradiation and orthotopically implanted them into nude mice (Fig. 6e). At 14 days post implantation, magnetic resonance imaging (MRI) showed decreased mean tumour volumes of FFX (50.58 mm^3^) and 8 Gy (32.43 mm^3^) pretreated groups compared with the control (376.52 mm^3^) in the epithelial PDAC subtype (Fig. 6e,f). Similarly, mesenchymal tumours showed a marginal decrease in mean tumour volume after FFX treatment (623.57 mm^3^) and a significant decrease after irradiation (155.93 mm^3^) compared with the controls (744.37 mm^3^) (Fig. 6e,f). Histological grading of implanted mesenchymal tumours was unchanged in comparison to implanted epithelial tumours. We noted that after 8 Gy radiation, 3 of 6 mice with epithelial tumours displayed an improved differentiation (Fig. 6g,h).

Although both in vitro and in vivo cytotoxic treatment regimens (FFX or 8 Gy) demonstrated specific responses depending on the transcriptional PDAC subtype, we next set out to determine whether individual organoid phenotypes display unique treatment vulnerabilities. Therefore, we performed a comprehensive phenotypic analysis of 915 epithelial and 1,257 mesenchymal single-cell-derived PDAC organoids exposed to FFX or 8 Gy treatments. In both transcriptional subtypes, we observe an emerging phenotype characterized by scattered organoids (red group) harbouring smaller, atypical shape and limited cell–cell contacts indicative of a toxicity-driven response (Fig. 6i). Importantly, we also identified specific changes in phenotype distribution upon treatment. For example, epithelial organoids of the cystic branched phenotype (brown group) were sensitive to both types of treatment (7.23% control to 1.16% after FFX and to 0 after 8 Gy) and failed to recover after the washout phase (Fig. 6i). Interestingly, the thick cystic/granular type (green group) was markedly decreased upon treatment (36.19% control to 9.3% after FFX and to 0 after 8 Gy); however, it rapidly recovered after FFX washout (19.28%). In contrast, TEBBO organoids (blue group) were essentially static under FFX treatment (17.42% control to 16.28% after treatment), while tree-like organoids (yellow group) were not affected and even increased in number (from 6.16% control to 10.46% after FFX). Both of these organoid phenotypes are FFX resistant but sensitive to radiotherapy (TEBBO 7.76% and tree-like 3.88%). Lastly, we document that the thick branched phenotype was the most resistant to both chemotherapy and radiation therapy (27.61% control to 25.58% after FFX and 23.3% after 8 Gy).

For the mesenchymal subtype, we observed that the firework organoids (purple group) were almost unaffected after treatment with FFX (from 48.08% control to 40.17%); however, this phenotype was markedly reduced after 8 Gy radiotherapy (9.37%). Conversely, the branched mesenchymal organoids (dark blue group) were severely affected by FFX (from 33.62% control to 19.64%) but more resistant to irradiation (21.87%). Remarkably, star-like organoids (middle blue group) were resistant to FFX and were even able to increase their relative numbers from 17.40% to 26.04%, while irradiation decreased their relative numbers (6.25%) accompanied by the appearance of a clump phenotype (light blue group) (Fig. 6i).

Taken together, we observe PDAC subtype-specific vulnerabilities and resistance to conventional cytotoxic treatments in organoids. Importantly, defined organoid phenotypes from the same parental PDAC line display distinct responses to treatment and this response can be different depending on the type of treatment (chemotherapy vs irradiation). These findings clearly indicate that tumour cell heterogeneity can be functionalized to study treatment resistance using the branching organoid assay.

### Defining phenotype-specific targeted-therapy vulnerabilities

**Fig. 7:**
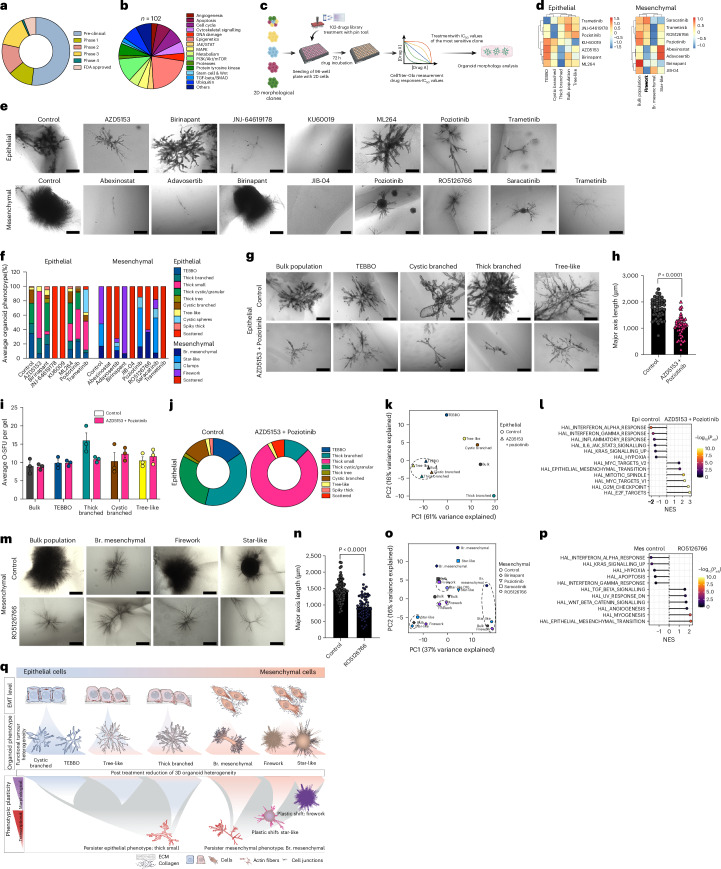
Targeted therapy reduces phenotypic heterogeneity via phenotypic reprogramming. Targeted therapy treatment and transcriptomic analysis of individual organoid phenotypes isolated from the E-mouse line ID: 9591 and the M-mouse line ID: 16992. **a**, Pie chart of the library design (*n* = 102 drugs) with the drug approval status: preclinical, phase 1, 2, 3, 4 and FDA approved. **b**, Pie chart of the specific targeted pathways by the 102 drugs. **c**, Schematic summary of the drug-treatment workflow; image created with BioRender.com. **d**, Heat maps of the *z*-score for specific drugs from the 102-drug screening of the 2D epithelial (4 phenotype clones and the bulk population from the mouse line ID: 9591, *n* = 2 individual experiments) and mesenchymal (3 phenotype clones and the bulk population from the mouse line ID: 16992, *n* = 2 individual experiments) cells. **e**, Brightfield imaging of bulk organoid morphologies post treatment with selective drugs using the IC_50_ values of the most sensitive 2D clones in 3D. Scale bars, 200 µm (mesenchymal treated with JIB-04 ), 500 µm (all others). **f**, Manual phenotypic analysis of epithelial bulk (*n* = 397 organoids from 3 individual experiments) and mesenchymal bulk (*n* = 246 organoids from 3 individual experiments) organoid populations after the selective treatment. Bar plot represents the average number of organoid phenotypes (%). **g**, Brightfield images of epithelial control (*n* = 334 organoids, 3 individual experiments) and combinatory treatment with AZD5153+Poziotinib (*n* = 321 organoids, 3 individual experiments) organoids from the bulk population, TEBBO, cystic branched, thick branched and tree-like phenotypes. Scale bars, 500 µm. **h**, Major axis length (µm) of bulk epithelial organoids as control (*n* = 51 organoids from 3 individual experiments) and organoids treated with AZD5153+Poziotinib (*n* = 49 organoids from 3 individual experiments). Graph represents mean ± s.e.m., unpaired two-tailed parametric *t*-test with Welch’s correction, two-tailed. **i**, O-SFUs per gel of control and AZD5153+Poziotinib-treated (in pink) epithelial organoid phenotypes; graph represents mean ± s.e.m. of 3 individual experiments. **j**, Manual phenotypic analysis of control and AZD5153+Poziotinib-treated bulk organoid populations of bulk epithelial organoids (*n* = 107 organoids). Pie chart represents the average number of organoid phenotypes (%). **k**, PCA analysis of the bulk RNA sequencing from control and AZD5153+Pozitionib-treated bulk, TEBBO, cystic branched, thick branched and tree-like organoids. Each dot represents the mean of 3 individual experiments. Dashed circles highlight the organoid phenotypes under the combinatory treatments. **l**, Dot plot of the GSEA comparing the epithelial control (from all phenotypes) and AZD5153+Poziotinib-treated (from all phenotypes) epithelial organoids. *P*_adj_, Benjamini–Hochberg adjusted *P* values. **m**, Mesenchymal control and RO5126766-treated organoid morphologies for the bulk population, branched mesenchymal, firework and star-like phenotypes. Scale bars, 500 µm. **n**, Major axis length (µm) of bulk mesenchymal organoids as control (*n* = 93 organoids from 3 individual experiments) and after treatment with RO5126766 (*n* = 67 organoids from 3 individual experiments). Graph represents mean ± s.e.m., unpaired two-tailed parametric *t*-test with Welch’s correction, two-tailed. **o**, PCA analysis of the bulk RNA sequencing from control and bulk, branched mesenchymal, firework and star-like organoids treated with Birinapant, Poziotinib, Saracatinib or RO5126766. In addition, we included an earlier time point for the star-like organoids (Day 10). Dashed circles highlight the organoid phenotype transitions under specific treatments. Each dot represents the mean of 3 individual experiments (except for the branched mesenchymal+RO5126766 with 1 replicate). **p**, Dot plot of GSEA comparing the mesenchymal control (from all phenotypes) with the RO5126766-treated (from all phenotypes) mesenchymal organoids. **q**, Graphical summary of the distinct epithelial and mesenchymal organoid phenotypes and how mono or combinational treatments can either plastically switch morphologies or reveal persister phenotypes. Br. mesenchymal, branched mesenchymal. Source data

**Extended Data Fig. 6:**
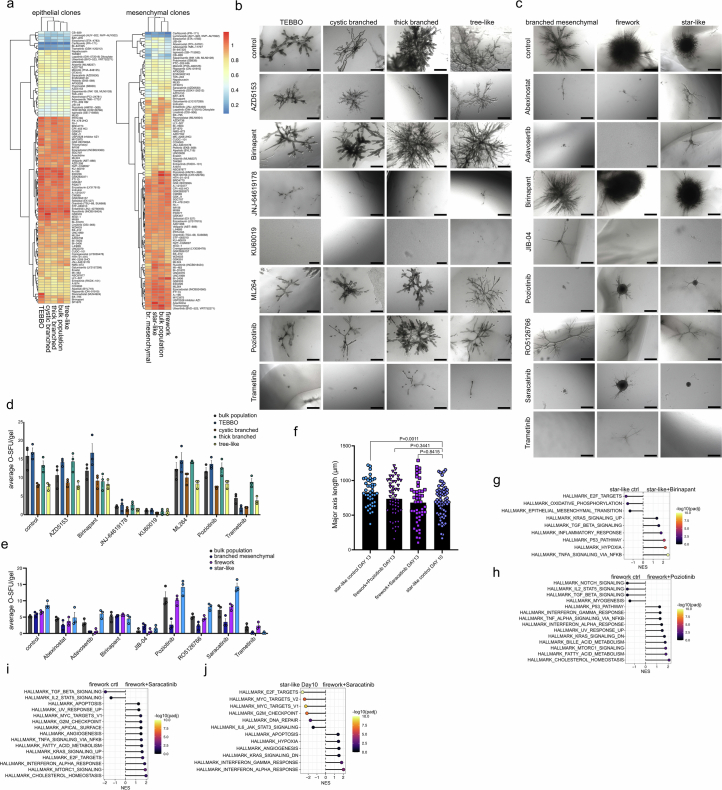
Pharmacotyping reveals unique organoid responses towards targeted therapies. All epithelial organoid phenotypes in Fig. 6 are derived from the mouse line ID: 9591 and the mesenchymal from the mouse line ID: 16992. **a** Heatmap of AUC values from epithelial (left) and mesenchymal (right) clones. **b** Representative organoid morphologies of epithelial 3D morphological clones: TEBBO, cystic branched, thick branched, and tree-like (from left to right) under control (DMSO treated) conditions or treated with the most sensitive IC_50_ values of: AZD5153, Birinapant, JNJ-64619178, KU60019, ML264, Poziotinib and Trametinib. All scale bars= 500 μm except for the TEBBO and thick branched phenotypes treated with KU60019, scale bars= 200 μm. **c** Representative organoid morphologies of mesenchymal 3D morphological clones: branched mesenchymal, firework-like and star-like (from left to right) under control conditions or treated with the most sensitive IC_50_ values of: Abexinostat, Adavosertib, Birinapant, JIB-04, Poziotinib, RO5126766, Saracatinib and Trametinib. All scale bars= 500 μm except for the firework phenotypes treated with Abexinostat, Adavosertib or JIB-04, scale bars= 200 μm. **d** Average Organoid structure formation units O-SFU/gel for the epithelial 3D morphological clones under the abovementioned (Extended Data Fig. 6b) treatment conditions. Graph represents mean±sem, from 3 individual experiments. **e** Average Organoid structure formation units O-SFU/gel for the mesenchymal 3D morphological clones under the abovementioned (Extended Data Fig. 6c) treatment conditions. Graph represents mean±sem, from 3 individual experiments. **f** Major axis length (in μm) of star-like control at Day 13 (n = 42 organoids), star-like control at Day 10 (n = 68 organoids), and firework organoids at Day 13 after treatment with Poziotinib (n = 53 organoids) or Saracatinib (n = 47 organoids). Graph represents mean±sem, unpaired two-tailed parametric t-test with Welch’s correction, two-tailed. **g** Dot plot of the GSEA from the mesenchymal star-like organoid phenotype control vs. Birinapant treated. **h** Dot plot of the GSEA from the mesenchymal firework organoid phenotype control vs. Poziotinib treated. **i** Dot plot of the GSEA from the mesenchymal firework organoid phenotype control vs. Saracatinib treated. **j** Dot plot of the GSEA from the mesenchymal star-like (Day 10) organoid phenotype control vs. the firework organoids treated with Saracatinib (star-like acquired phenotype, at Day 13). **g-j** NES= normalized enrichment score, padj=Benjamini-Hochberg adjusted p-values. Source data

Since specific organoid phenotypes within both transcriptional PDAC subtypes (for example, TEBBO after FFX, thick branched after both FFX/8 Gy, firework/star-like under FFX and branched mesenchymal after 8 Gy) display resistance to conventional cancer treatments, we next sought to identify targeted treatment strategies to eliminate specific ‘persister’ organoid phenotypes. We employed a drug library of 102 compounds targeting a wide range of cellular processes, including DNA damage, apoptosis, cell cycle, receptor tyrosine kinases signalling, intracellular kinases signalling, cytoskeleton formation as well as epigenetic regulators, in different stages of clinical implementation—from preclinical to US Food and Drug Administration (FDA) approval (Fig. 7a,b). This handpicked library has been tested as part of several previous PDAC drug-testing efforts including longitudinal drug testing using PDAC patient-derived organoids^41^, in the context of sensitizing strategies to Ras-Raf-Mek Erk pathway inhibition^42^ as well as in combinatorial drug screens^43^. First, we generated and expanded stable 2D clones that give rise to all major phenotypical subclusters (2D-E TEBBO, 2D-E cystic branched, 2D-E thick branched, 2D-E tree-like, 2D-M branched mesenchymal, 2D-M firework, 2D-M star-like) (see also Extended Data Fig. 4e,f) and subjected these clones to the drug library (Fig. 7a–c and Extended Data Fig. 6a). We determined the response to treatment by quantifying the area under the curve (AUC) (Extended Data Fig. 6a) and further selected specific drugs exerting the most pronounced differential responses between the phenotype clones (Fig. 7d). Importantly, we observe that clones derived from the same parental lines respond very heterogeneously to targeted therapies, especially in terms of viability of the different clones that gave rise to distinct phenotypes, providing insight into the impact of intra-cell-line heterogeneity on drug resistance (Extended Data Fig. 6a). We then tested this subset of drugs in the branching organoid system to determine organoid phenotype-specific vulnerabilities by administering the IC_50_ values of the most sensitive clone, with the overarching goal of reducing phenotypic heterogeneity (Fig. 7d).

In the bulk epithelial organoids, treatment with JNJ-64619178 (PRMT5 inhibitor) and KU60019 (ATM inhibitor) severely impacted the morphology, resulting in a complete scattered phenotype. In addition, Trametinib (MEK1/2 inhibitor), Poziotinib (pan-HER inhibitor) and AZD5153 (BET/BRD4 inhibitor) resulted in formation of a new phenotype, the thick small organoid with ML264 (KLF5 inhibitor) also having an increased proportion of thick small organoids. Birinapant (SMAC mimetic), on the other hand, had little effect on the epithelial organoid morphologies (Fig. 7e,f). To validate the specificity of our approach, we also tested individual organoid phenotypes treated with the same compounds. For example, the thick branched epithelial phenotype is highly responsive to AZD5153 (BET/BRD4 inhibitor) treatment, indicated by a phenotypic switch towards more thick small and even scattered phenotypes without decreasing the O-SFU. In contrast, the TEBBO, cystic branched and tree-like phenotypes appear largely unaffected by AZD5153 (BET/BRD4 inhibitor) treatment (Extended Data Fig. 6b,d). Conversely, Poziotinib (pan-HER inhibitor) affected mostly the TEBBO and cystic branched organoids, whereas the thick branched organoids remained unaffected. Moreover, the KLF5 inhibitor, ML264, had a much stronger impact on the TEBBO organoid phenotype, including a reduction in size and terminal end bud formation (Extended Data Fig. 6b). To overcome heterogeneity-driven resistance in epithelial PDAC cells and test our overarching strategy of state-gating and state-targeting PDAC therapy, we chose to combine two highly potent drugs from our pharmacological screen, AZD5153 (BET/BRD4 inhibitor) and Poziotinib (pan-HER inhibitor) (Fig. 7g). Interestingly, combinatorial treatment with AZD5153 and Poziotinib revealed an enrichment of a major resistant morphological phenotype, the thick small organoid, with significantly reduced size (major axis length, control = 1,753.15 µm vs treated = 1,067.48 µm) (Fig. 7h), accounting for 79.6% of the bulk population vs 59.6 or 42.6% in the monotreatment with AZD5153 or Poziotinib, respectively (Fig. 7f,j). After treatment of both the bulk population and individual organoid morphologies, phenotypes were shifted towards this resistant phenotype without affecting their O-SFU (Fig. 7i).

On a transcriptomic level, we observe that while both epithelial bulk and individual organoid phenotypes are distinct from one another in control conditions (spread along PC2), all models respond to AZD5153 and Poziotinib (combinatory) treatment and converge towards a similar phenotype (Fig. 7k). Gene set enrichment analysis revealed that epithelial organoids after combinatory treatment with AZD5153 and Poziotinib (thick small phenotypes) are enriched for EMT, Myc and proliferation, while control organoids maintained a strong inflammatory, hypoxic and Kras signature (Fig. 7l).

We next focused on the mesenchymal subtype since patients with undifferentiated PDAC display increased resistance to chemotherapy and reduced overall survival^3^. Moreover, as reported above, mesenchymal PDAC organoids are less sensitive to FOLFIRINOX compared with epithelial PDAC organoids. In the mesenchymal cluster, we tested 8 different drugs and evaluated the response of organoid phenotypes (Fig. 7d,e).

Treatment of the bulk organoid population with Abexinostat (pan-HDAC inhibitor), Adavosertib (Wee1 inhibitor), JIB-04 (pan-selective Jumonji histone demethylase inhibitor), RO5126766 (dual MEK/RAF inhibitor) and Trametinib (MEK1/2 inhibitor) resulted in the formation of mostly scattered organoids (60% in the RO5126766, 72% in the Adavosertib and 100% for Abexinostat, Trametinib and JIB-04) (Fig. 7f). Importantly, Birinapant (SMAC mimetic) had a strong impact on specific phenotypic clones, by morphologically shifting the star-like phenotype into a firework phenotype without affecting the firework organoid morphology (Fig. 7e,f and Extended Data Fig. 6c,e). In contrast, the previously mentioned Poziotinib (pan-HER inhibitor) and Saracatinib (Src inhibitor) had growth inhibitory effects on star-like organoids but virtually eliminated all branched mesenchymal organoids (Fig. 7f and Extended Data Fig. 6c). Interestingly, firework organoids transformed into an immature, relatively smaller phenotype corresponding to the star-like phenotype on Day 10, underscoring the plasticity of specific organoid phenotypes. In detail, firework organoids under treatment with Poziotinib/Saracatinib became star-like with a reduced major axis length: firework+Poziotinib = 735.81 µm, firework+Saracatinib = 681.54 µm, star-like control (Day 13) = 829.85 µm and star-like Day 10 = 691.91 µm (Extended Data Fig. 6c,f).

The branched mesenchymal phenotype was the most resistant to RO5126766 (dual MEK/RAF inhibitor) and treatments with this inhibitor consolidated all phenotypes into a thinner and smaller version of the branched mesenchymal phenotype (bulk controls = 1,428.76 µm vs treated = 957.10 µm) (Fig. 7m,n).

To assess whether these pronounced morphological changes also result in corresponding transcriptional reprogramming, we performed transcriptomic analysis of control and treated mesenchymal phenotypes. On a global PCA level, star-like organoids, which assumed a firework morphology upon treatment with Birinapant, did not cluster with the firework control but remained close to the original star-like state, exerting only minor gene-expression differences (Fig. 7o and Extended Data Fig. 6g). Similarly, the firework organoids treated with Poziotinib (induced star-like morphology) clustered together with the control (firework) organoids, indicating no major transcriptional effects (Fig. 7o and Extended Data Fig. 6h). Interestingly, firework organoids treated with Saracatinib (also induced star-like morphology) underwent a slight shift towards the immature (Day 10) star-like organoids (Fig. 7o and Extended Data Fig. 6i,j). Most remarkably, upon RO5126766 treatment, all mesenchymal organoids morphologically shifted towards the branched-mesenchymal phenotypes and transcriptionally converged (consolidated along PC2) (Fig. 7o). Gene set enrichment analysis of the mesenchymal phenotypes revealed that the RO5126766-induced branched mesenchymal organoids were enriched for EMT and myogenesis, while control phenotypes expressed interferon, Kras signalling as well as hypoxia signatures (Fig. 7p).

Taken together, these data demonstrate that our branching organoid assay allows capturing of PDAC morphological heterogeneity, and the combination of phenotypic mapping with targeted therapy reveals highly diverse intratumoural treatment responses as well as unique phenotype-specific vulnerabilities. Epithelial organoids display more heterogeneous phenotypes and require combinational targeted therapy as a cell-state-gating strategy. In contrast, mesenchymal organoids plastically shift their morphology to adapt to treatment. Interestingly, upon defined targeted therapies in mesenchymal organoids (for example, RO5126766), or the combinatorial treatment in epithelial organoids, this consolidating phenomenon is traceable phenotypically (morphologically and transcriptionally) (Fig. 7q).

### Functionalizing PDAC patient-derived organoids to model intratumoural heterogeneity

**Fig. 8:**
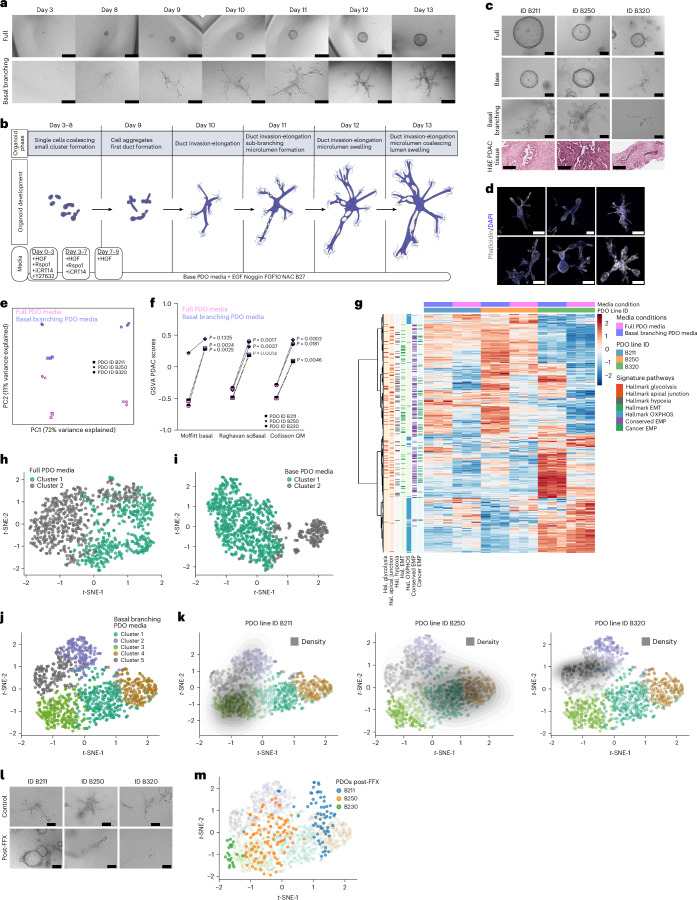
Patient-derived organoids develop heterogeneous phenotypes in basal branching PDO media. **a**, Daily imaging of single-cell-derived organoids over the course of 13 days of development (here representing Days 3 and 8–13). The PDOs were cultured in full PDO media (top; *n* = 342 organoids) and in basal branching PDO media (bottom; *n* = 573 organoids). Scale bars, 200 µm (full PDO media); 200 µm (Days 3–11) and 500 µm (from Day 12 onwards) for the basal branching PDO media. **b**, Graphical summary of the organoid development in the basal branching PDO media with the media composition and the different developmental phases. **c**, Characteristic PDO organoid morphologies (*n* = 3 PDO lines) cultured in different conditions: full PDO media (top), base PDO media (middle), basal branching PDO media (bottom) and H&E staining of the corresponding primary tumours. Scale bars, 100 µm (PDO line ID B320 cultured in full PDO media and base PDO media), 200 µm (all others). **d**, Confocal IF imaging of phalloidin (white) and DAPI (blue). Scale bar, 200 µm, illustrating the different morphologies the PDO line ID B250 displays in basal branching PDO media. **e**, PCA of bulk RNA sequencing for the PDO lines (IDs: B211, B250 and B320) in full PDO media (pink colour) and basal branching PDO media (lilac colour). **f**, Gene set variation analysis (GSVA) of the basal/quasi-mesenchymal profile^48–50^ of PDO lines cultured in full PDO media and basal branching PDO media. Unpaired two-tailed parametric *t*-test with Welch’s correction, two-tailed. **g**, Heat map of bulk RNA sequencing for the PDO lines (IDs: B211, B250 and B320) for the hallmarks: Glycolysis, Apical Junction, Hypoxia, EMT, Oxidative phosphorylation, EMP and Cancer EMT characterizing the individual PDOs cultured in full PDO media or in basal branching PDO media. **h**, *K*-means clustering of the image-derived features of unseen data set of *n* = 832 organoids reveals 2 distinct morphological clusters when PDOs were cultured in full PDO media. **i**, *K*-means clustering of the image-derived features of unseen data set of *n* = 834 organoids reveals 2 distinct morphological clusters when PDOs were cultured in base PDO media. **j**, *K*-means clustering of the image-derived features of unseen data set of *n* = 928 organoids reveals 5 distinct morphological clusters when PDOs were cultured in basal branching PDO media. **k**, Individual PDO line (ID: B211 (left), ID: B250 (middle), ID: B320 (right)) grown in basal branching PDO media, with morphological heterogeneity as visualized by density overlays superimposed on the imaged-derived clusters. The overlays indicate which cell lines correspond to which cluster. **l**, Characteristic morphologies of organoids grown in basal branching PDO media as control or after pre-treatment with FFX IC_50_ values. Scale bars, 200 µm. **m**, Clustering of PDOs (superimposed on the *K*-means clustering from **j**) after pre-treatment with IC_50_ values of FFX and then culturing in basal branching PDO media. Source data

**Extended Data Fig. 7:**
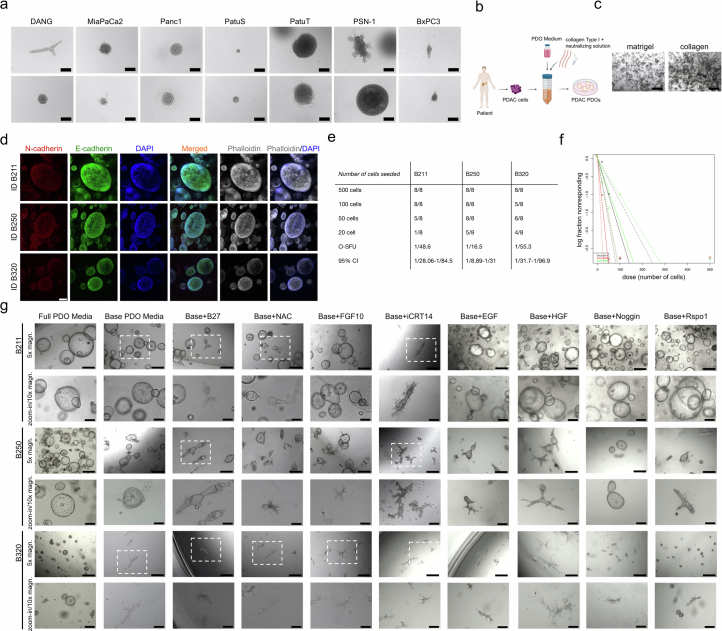
Defining the composition of the basal branching PDO media to induce branching morphogenesis in PDOs. **a** Representative organoid morphologies of established PDAC lines: DANG, MiaPaCa2, Panc1, PatuS, PatuT, PSN-1 and BxPC3 (from left to right) grown inside floating collagen gels in their established media culture conditions: (either RPMI or DMEM + 10% FBS) (n = 2035 total organoids). All scale bars= 200 μm. **b** Schematic representation of the PDO generation. Image created with BioRender.com. **c** Organoid morphological resemblance in Matrigel and Collagen matrix grown conditions. Scale bars= 500 μm. **d** Confocal IF imaging of PDOs grown in floating collagen gels for E-cadherin (green), N-cadherin (red), DAPI (blue) and phalloidin (white), scale bars= 100 μm. **e** Extreme Limiting Dilution Analysis (ELDA) of different PDO lines. **f** Plot of the log fraction of nonresponding wells (empty of organoids) versus the number of the seeded cells. **g** Representative organoid morphologies of the PDO lines ID B211, B250 and B320 in Full PDO Media, Base PDO Media and Base PDO Media with addition of 1% B27, 500 µM N-acetyl cysteine (NAC), 25 ng FGF10, 50 ng EGF, 10 ng HGF, 25 ng Noggin, 25 ng Rspondin1 or 5 µM iCRT14 (n = 350 organoids for B211, n = 470 organoids for B250, n = 413 for B320). Top panel is a 5x magnification, scale bar= 500 μm and bottom panel is a zoom-in 10x image or higher magnification (10x) images, scale bar= 200 μm for each individual PDO line.

**Extended Data Fig. 8:**
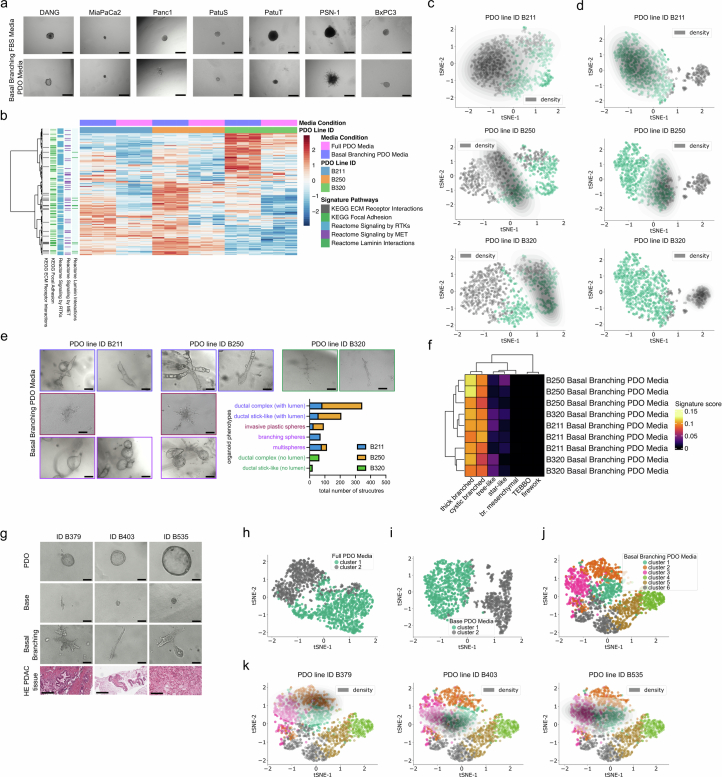
Capturing heterogeneous branching in PDO phenotypes. **a** Representative organoid morphologies of established PDAC lines: DANG, MiaPaCa2, Panc1, PatuS, PatuT, PSN-1 and BxPC3 (from left to right) grown inside floating collagen gels in two different media culture conditions: the established media conditions containing 10% FBS (top) and the Basal Branching PDO Media (bottom) (n = 113 organoids in the 10% FBS Media and 106 organoids in the Basal Branching PDO Media). All scale bars= 500 μm except from the PatuS and BxPC3 organoids where the scale bars= 200 μm. **b-f** Analyses of the following PDO line IDs: B211, B250, B320. **b** Heatmap of bulk RNA sequencing for different pathways associated with the extracellular matrix organization and rearrangement. **c** Individual PDO line morphological heterogeneity in Full PDO Media as visualized by density overlays superimposed on the imaged-derived clusters. The overlays indicate which cell lines correspond to which cluster. **d** Individual PDO line morphological heterogeneity in Base PDO Media as visualized by density overlays superimposed on the imaged-derived clusters. The overlays indicate which cell lines correspond to which cluster. **e** Major morphologies found in PDOs cultured in Basal Branching PDO Media. Color coding implies the hierarchical relation of the super families and manual morphological clustering of the total number of organoids, n = 916 organoids. Scale bars= 200 μm. **f** Transcriptional correlation scoring of PDOs growing in Basal Branching PDO Media to murine organoid phenotype signatures. **g** Characteristic PDO organoid morphologies of additional PDO lines (n = 3 patients, PDO line IDs: B379, B403, B535) cultured in different conditions: Full PDO Media (top), Base PDO Media (middle), Basal Branching PDO Media (bottom) and HEs of the corresponding PDAC tissues. All organoid scale bars= 100 μm, HEs of the PDAC tissues scale bars= 200 μm. **h** K-means clustering of the image-derived features of an additional unseen data set from the PDO line IDs: B379, B403, B535 (n = 531) organoids integrated with the original data from Fig. 8h reveals 2 distinct morphological clusters when PDOs are cultured in Full PDO Media. **i** K-means clustering of the image-derived features of an additional unseen data set from the PDO line IDs: B379, B403, B535 (n = 349 organoids) integrated with the original data from Fig. 8i reveals 2 distinct morphological clusters when PDOs are cultured in Base PDO Media. **j** K-means clustering of the image-derived features of an additional unseen data set from the PDO line IDs: B379, B403, B535 (n = 783 organoids) integrated with the original data set from the Fig. 8j reveals 6 distinct morphological clusters when PDOs are cultured in Basal Branching PDO Media. **k** Individual PDO line (ID: B379-left, ID: B403-middle, ID: B535-right) morphological heterogeneity in Basal Branching PDO Media as visualised by density overlays superimposed on the imaged-derived clusters (Extended Data Fig. 8j). The overlays indicate which cell lines correspond to which cluster. Source data

To apply our findings to human disease, we tested the organoid formation capacity and morphologies of 7 established, commercially available human PDAC cell lines (DANG, MiaPaCa2, Panc1, PatuS, PatuT, PSN-1, BxPC3). Although it was demonstrated that some of these established human PDAC cell lines (MiaPaCa2 and Panc1) display inherent heterogeneity on a genomic and transcriptomic level^44^, in our branching organoid assay, this heterogeneity failed to translate into phenotypic diversity, as all cell lines tested mostly formed spheres (Extended Data Fig. 7a). To assess whether this impaired morphogenesis represents an artefact of these conventional PDAC cell lines, we next tested PDAC patient-derived organoids (PDOs). Of note, PDOs recapitulate tumour histology after implanting into mice and retain genomic alterations^45^ of the parental tumour as well as the transcriptional intratumoural heterogeneity^46^. PDOs were generated from endoscopic ultrasound-guided fine-needle aspiration biopsies or surgical resection specimens^47^ and cultured inside a Matrigel ECM (Extended Data Fig. 7b,c). PDOs were then embedded into floating collagen gels and full PDO media were added (Extended Data Fig. 7b,c). Importantly, in contrast to primary murine PDAC cells and similar to the established human PDAC cell lines, PDOs display only very little phenotypic variation, mostly forming cystic spherical tumour organoids (Extended Data Fig. 7c,d). We have demonstrated previously that the formation of branching organoids is achieved only when combined with the right matrix (biophysical properties of the matrix) and media supplementation^14^. Therefore, in addition to culturing PDOs in floating gels, we sought to alter the media composition. In fact, the full PDO media contain many factors known to enhance epithelial (classical) differentiation and inhibit branching morphogenesis, such as A83-01(TGFβ-RI inhibitor), in PDAC organoids (see also Fig. 2d). Besides A83-01, we stripped the media of other supplements such as cholera toxin, bovine pituitary extract (BPE), R-spondin conditioned media and Wnt3a, all harbouring pleiotropic or inhibitory effects on branching morphogenesis. We termed this reduced and simplified media base PDO media (see Methods). We then systematically tested media compositions facilitating PDO branching and phenotypic heterogeneity. Specifically, we assayed growth factors B27, FGF10, EGF, HGF, Noggin and Rspondin1, as well as the small molecules or inhibitors NAC and iCRT14 (Extended Data Fig. 7g), titrated in a timely fashion, leading to branching morphogenesis in PDOs (Fig. 8a,b and see Methods). Next, we compared this newly created branching PDO media to the established full PDO media. In detail, we seeded clonal densities of PDOs (Extended Data Fig. 7e,f) into floating collagen gels. PDOs were imaged for 13 days (Fig. 8a), revealing minimal morphological changes under the full PDO media conditions, with organoid expansion and lumen swelling. In contrast, when the same PDO lines were cultured in the branching PDO media, PDOs underwent multiple phases of ductal invasion–elongation, branching and sub-branching events, microlumen swelling and coalescing into a continuous lumen connecting the organoid (Fig. 8a,b). Interestingly, within our PDO panel, one line (ID: B320) was able to break symmetry and undergo branching morphogenesis even when cultured in base PDO media conditions (Fig. 8c). PDOs cultured in the branching PDO media formed complex tubular structures (Fig. 8d) reminiscent of the primary tumour architecture (Fig. 8c). Importantly, the abovementioned established human PDAC cell lines (DANG, MiaPaCa2, Panc1, PatuS, PatuT, PSN-1, BxPC3) displayed no branching phenotype under these conditions, indicating impaired plasticity (Extended Data Fig. 8a).

Next, we performed transcriptomic analysis of PDOs grown in full PDO media vs branching PDO media, revealing profound differences in major transcriptional programmes (Fig. 8e), with PDOs cultured in the branching PDO media overexpressing signatures of glycolysis, apical junctions, hypoxia and EMT/EMP versus an upregulation of oxidative phosphorylation signatures in the full PDO media conditions (Fig. 8g). In addition, gene-expression programmes of PDOs in branching conditions display vigorous interactions and rearrangement of the extracellular matrix, accompanied by the formation of focal adhesions, ECM receptor interactions, laminin interactions and signalling by MET/receptor tyrosine kinases (Extended Data Fig. 8b). Of note, the pronounced EMT induction and ECM remodelling observed in the branching PDO media is accompanied by increased signatures of the basal PDAC subtype^48–50^ (Fig. 8f). We therefore hypothesized that this basal branching PDO media might be key for the acquisition of EMT/EMP traits in PDOs to facilitate EMP-driven phenotypic diversity and eventually model ITH.

### PDOs grown in basal branching PDO media display heterogeneous organoid morphologies

To test this hypothesis, we analysed phenotypic organoid diversity of PDOs in standard conditions (full PDO media), reduced media (base PDO media) and the basal branching PDO media, and clustered organoids morphologically (*n* = 2,594 individual PDOs) (Fig. 8h–j). As expected, both conditions, the full and the base PDO media, revealed limited morphological heterogeneity, displaying only two distinct phenotypic clusters in all PDOs tested (Extended Data Fig. 8c,d). In the case of the full PDO media, these two clusters were derived from the size difference of these PDOs, with cluster 1 (green colour) representing larger PDOs and cluster 2 (anthracite colour) representing smaller PDOs (Extended Data Fig. 8c). When PDOs were cultured in the base PDO media, a clearer separation of the organoids appeared, with 2 major clusters: one for the PDOs that maintained their cystic morphology (PDO line IDs: B211 and B250, green cluster) and another for the PDO line ID: B320 (anthracite colour) that underwent branching morphogenesis (Extended Data Fig. 8d). In stark contrast, the basal branching PDO media facilitated increased intra-organoid heterogeneity with 5 distinct morphological clusters (Fig. 8j,k). When we superimposed an additional panel of 3 PDO lines (PDO line IDs: B379, B403, B535) (Extended Data Fig. 8g), we observed an increase in phenotypic diversity by one additional cluster, with a total of 6 morphologic clusters present in the branching PDO media (Extended Data Fig. 8h–k). Similar to the murine PDAC branched organoids, we observed heterogeneous branching phenotypes ranging from complex ductal phenotypes to invasive plastic spheres (Extended Data Fig. 8e and see Methods). In line with this, when comparing PDO transcriptional profiles to the murine organoid phenotype signatures, we observed an enrichment for the thick branched and cystic branched signatures (Extended Data Fig. 8f).

As we have done in the murine system, we next tested whether our branching organoid assay is also able to capture phenotypic changes under treatment-imposed pressure in the PDAC patient-derived model and whether standard-of-care chemotherapy affects intra-organoid heterogeneity. To this end, we pretreated PDOs with their respective IC_50_ values of FOLFIRINOX (FFX) and then seeded them into branching organoid conditions using the basal branching PDO media. Indeed, FFX treatment drastically reduced branching capacity, with PDO line B320 being unable to undergo morphogenesis or form any coherent organoid structures (Fig. 8l). PDOs B211 and B250 still formed multicellular structures albeit less complex, with only PDO B250 being able to break symmetry and form tubular structures (Fig. 8l). Importantly, superimposing the post-treatment PDO morphologies onto MOrPHeMap demonstrated a clear reduction in organoid heterogeneity, with PDO B211 consolidating into one cluster, PDO B250 mostly limited to two clusters and PDO B320 clustering outside the map, indicating sustained treatment effects and a reduction in heterogeneity (Fig. 8m). In conclusion, even a rather conventional therapeutic approach, such as polychemotherapy, exerts specific effects on defined organoid phenotypes, phenotypic variability and branching capacity of PDOs.

Taken together, these data indicate that culturing PDOs in basal branching PDO media conditions allow us to capture phenotypic organoid heterogeneity in a clinically meaningful PDAC patient-derived model system. Importantly, this heterogeneity can be altered upon experimental treatment, providing a functional system to screen for state-gating and state-targeting PDAC therapies.

## Discussion

Phenotypic plasticity refers to the ability of cancer cells to acquire different identities within a given phenotypic spectrum^12^. In non-cancerous conditions, cellular plasticity is orchestrated carefully in complex biological processes such as embryonic development and tissue regeneration. In cancer, tumour cells are able to (re-)activate plasticity programmes to block or revert to terminal- and trans-differentiation processes such as the EMT, a key process in carcinogenesis^13,33,51,52^. Importantly, reprogramming to become phenotypically plastic fuels drug refractory states, and thereby, resistance to therapy^53^. Therefore, unlocking this phenotypic plasticity is considered an emerging hallmark of cancer^13^. EMT is the most studied mechanism of phenotype switching and plasticity^53,54^, and pancreatic cancer cells are notoriously plastic^55^.

**Extended Data Fig. 9:**
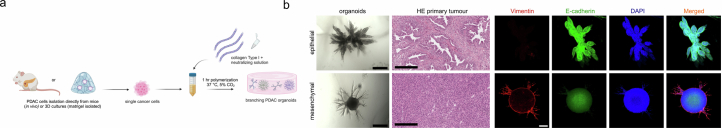
Culturing of branching epithelial and mesenchymal organoids directly in 3D matrices. **a** Schematic representation of organoid generation directly from *in vivo* or 3D *in vitro* cultures (Matrigel), image created with BioRender.com. **b** Epithelial (top) and mesenchymal (bottom) collagen gel grown organoids. Corresponding HEs of the primary tumours. Confocal IF imaging of E-cadherin (green), Vimentin (red), DAPI (blue). Scale bars= 500 μm (bright field), 200 μm (HEs), 200 μm (IF).

To provide an experimental system to functionalize phenotypic tumour plasticity and heterogeneity, we present a 3D organoid model of pancreatic cancer, from mouse and human tissue, capturing the entire EMT spectrum morphologically and on the molecular level. Branching organoids inside collagen type-I gels are able to remodel the surrounding collagen matrix (collagen fibre alignment) and educate the ECM environment by secreting ECM proteins such as collagens, laminins and fibronectin^14^. This tumour cell intrinsic process of matrix remodelling and education in our organoid cultures might explain the minimal effects of extrinsic manipulation of these key parameters of the ECM microenvironment. Importantly, the phenotypic diversity of branching PDAC organoids is not restricted to primary tumour cells derived from 2D culture conditions, but morphogenesis programmes driving phenotypic organoid diversity are also present in tumour cells previously propagated in 3D matrices, such as Matrigel, and most notably, when isolated directly from in vivo, yielding similar branching organoid phenotypes (Extended Data Fig. 9a,b).

Mechanistically, we identified that branching morphogenesis in both epithelial and mesenchymal transcriptional clusters of PDAC organoids depends on an EMT-inducing pathway, the canonical TGFβ pathway, to acquire phenotypic diversity. Aberrant expression or dysregulation of the TGFβ pathway leads to reduced or inhibited branching morphogenesis. In agreement with our data, a previous study demonstrated that tube morphogenesis rather than sphere formation in PDAC relies on active TGFβ signalling^56^. In addition, we used TGFβ-induced EMT to reveal two distinct epithelial populations: one with long plasticity memory that can revert to the phenotype of the cell of origin and another with short plasticity memory that maintains its mesenchymal morphological features after withdrawal of the EMT stimulation.

So far, a number of studies have identified the pre-existence of an EMT continuum in cancers, with epithelial, intermediate EMT, quasi-mesenchymal and mesenchymal transition states that can be stable over time and drive ITH^5,27,57^. In this study, we identified the emergence of multiple organoid phenotypes derived from the same parental tumours and correlated pre-existing EMT tumour cell heterogeneity and diverse tumour cell states with distinct organoid phenotypes.

When transplanting tumour cells from distinct organoid phenotypes, we were able to detect remarkable differences in in vivo tumour morphogenesis phenocopying the individual organoid morphologies. Interestingly, organoid cultures post transplantation recapitulate the phenotype of the organoid of origin to a high degree, indicating a stable tumour cell state. A previous study proved that tumour cells from different EMT transition states had similar propagating efficacies but distinct plasticity levels (differentiating in the primary tumour) as well as different metastatic capacities (lung colonization)^27^. Interestingly, we also observed no differences in tumour initiation (engraftment) between organoid phenotypes in our study; however, the tree-like- and thick branched-derived tumours (epithelial PDAC subtype) as well as the star-like-derived tumours (mesenchymal PDAC subtype) harbour increased metastatic capacities, underscoring functional differences in individual tumour cell states represented by distinct organoid phenotypes.

A recent study employing colorectal cancer patient-derived organoid cultures and subsequent phenotyping based on organoid size and cyst formation versus solid morphology indicates that these basic organoid phenotypes are driven by defined signalling pathways^58^. In another study using 2D non-small-cell lung cancer (NSCLC) cell lines, neural net algorithms were used to create reference maps of lung cancer EMT and MET transition states^59^. Here we developed an organoid technology that allows PDAC organoids to acquire complex branched phenotypes reflecting the individual EMT state accompanied by unique therapeutic vulnerabilities. To amplify our efforts in generating a comprehensive PDAC organoid landscape, we developed an unbiased mapping technology, termed MOrPHeMap (Morphologic Organoid Phenotypic Heterogeneity Mapping), which is able to identify distinct organoid morphologies and determine the phenotypical spectrum within individual organoid lines. Importantly, this phenotypic heterogeneity is not defined by the tumour driver mutation but by transcriptional programmes (for instance, the EMT status) of the individual tumour cell. Collectively, MOrPHeMap provides a unique approach to capture, quantify and functionalize dynamic phenotypic changes evolving upon perturbation, such as targeted therapies, and thereby opening new avenues to explore state-gating and state-targeting treatment strategies. For instance, epithelial phenotypes display impaired regeneration after standard-of-care polychemotherapy while exhibiting moderate resistance to irradiation. In contrast, mesenchymal phenotypes behave inversely, exhibiting resistance to FOLFIRINOX but high vulnerability to radiation therapy, and these differential treatment responses are also present in vivo. This phenomenon not only holds true for organoids derived from distinct transcriptional subtypes of PDAC (such as classical and basal-like) but also between individual organoid phenotypes from these subtypes.

In a recent review, Hydra, the mythological, nine-headed monster from ancient Greece, was used as an analogy for treatment response patterns in cancer and underlying molecular heterogeneity^60^. In contrast to Heracles who cut off all nine heads to defeat the monster, we propose a strategy to first consolidate the phenotypes into one ‘persister’ organoid phenotype which can subsequently be targeted. To this end, we employed an anticancer drug screen and identified candidates that specifically target our morphological clusters. In detail, we revealed that morphological clones from mesenchymal PDAC exhibit a more plastic behaviour with rapid morphological shifts under monotreatment as seen when using Birinapant (SMAC mimetic) or Poziotinib (pan-HER inhibitor). Firework organoids treated with Saracatinib (Src inhibitor) plastically acquire a star-like organoid morphology and transcriptionally cluster closer to immature star-like organoids. Most notably, with the highly effective RO5126766 (dual MEK/RAF inhibitor) treatment, all mesenchymal organoid phenotypes are reprogrammed (morphologically and transcriptomically), converging into a persistent organoid phenotype. In contrast, epithelial PDAC organoids are more heterogeneous, displaying increased morphologic diversity compared with mesenchymal PDAC organoids. To consolidate these heterogeneous populations, we employed combinatory treatments, revealing an epithelial ‘persister’ phenotype. Altogether, the combination of phenotypic mapping with targeted therapy reveals highly diverse intratumoural treatment responses as well as unique phenotype-specific vulnerabilities. In addition, we are able to capture dynamic phenotypic changes in plastic cancerous cell behaviour in an unprecedented fashion. Combining our understanding of transcriptional programmes governing individual organoid phenotypes and phenotypic shifts, we propose to eliminate intra-tumour cell heterogeneity, overcome plastic phenotype-driven drug resistance and target persistent clones^61^.

To translate these findings to human PDAC, we next tested PDOs^45^ in our system. Initially, when PDOs were tested in our branching organoid system, they failed to undergo branching morphogenesis and displayed little morphological variation. From the first description of pancreatic organoids^62^ and pancreatic tumour organoids^63^, cells have been predominantly cultured in Matrigel under media conditions that enrich for differentiated/epithelial subtypes. For this reason, we developed culture conditions permissive to dynamic alterations in cellular differentiation. In detail, we provided collagen type-I as floating gels and reconstructed media conditions to generate the basal branching PDO media. In our first step towards generating the basal branching PDO media, we cultured our PDOs in reduced media, the base PDO media in which cells displayed resistance to the absence of Wnt signalling ligands, as has been previously reported^64^. The basal branching PDO media not only enhances a more basal-like PDAC phenotype by upregulating the EMP profile of organoids, but also allows them to strongly interact with their surrounding extracellular matrix^65^, a process that has been identified as a necessary step to undergo branching morphogenesis^14^, thus generating heterogeneous morphological phenotypes. Previous studies already associated the classical media composition with dynamic changes in the heterogeneous transcriptional profiles of PDOs from single cell Basal in vivo towards single cell Classical signatures in vitro (when cultured as organoids), and identified that KRAS amplification alone was not sufficient to maintain a basal-like phenotype^49^. It yet remains to be proven whether the basal branching PDO media can indeed enhance specific pre-existing subpopulations that undergo branching morphogenesis and display heterogeneous phenotypes, as classical and basal-like cells can co-exist in PDOs^46,66^, or whether these changes are more global, affecting the entire PDO population. In the future, co-clinical testing will be critical to determine the prognostic value of our model system, as the media composition is known to influence not only the organoid transcriptome but also the response to chemotherapy^67^. As we previously demonstrated, PDOs generated before and after neoadjuvant chemotherapy in patients can dynamically change their transcriptional profile without clonal selection^41^. Here, when PDOs were treated with FOLFIRINOX and reseeded in floating collagen gels supplemented with the basal branching PDO media, PDOs displayed reduced branching and formed predominant cystic phenotypes, indicating reduced morphological heterogeneity.

In summary, we have shown that pre-existing EMP or increasing EMP levels are essential to generate heterogeneous branching organoid populations to model intratumoural heterogeneity in vitro. Combining the two technologies, namely, the branching organoid assay and MOrPHeMap, we can capture the mouse and human PDAC organoid landscape and the dynamic phenotypic changes occurring upon multimodal treatment. With the power that cancer organoids possess as miniature tumour avatars^68^, our study paves the way to using advanced organoid models to decipher phenotypic heterogeneity and ITH-driven treatment resistance in PDAC.

## Methods

### Study approval and clinical samples

Experiments involving human material were designed according to the Declaration of Helsinki and conformed to the Department of Health and Human Services Belmont Report. For the generation of patient-derived organoids, we used PDO lines either previously described^41,69^ or newly generated from patients with PDAC, upon acquiring their written consent approved by the ethics review board of the Klinikum rechts der Isar der TUM, School of Medicine and Health, Technical University of Munich (Institutional Review Board project nos. 207/15, 1946/07, 330/19S, 181/17S, 5542/12 and 80/17S).

### Animal experiments

#### Orthotopic implantation of organoid phenotypes in a syngeneic mouse model

For orthotopic transplantation of mouse PDAC organoid cultures, a single-cell suspension^47,63,70^ of 2,500 cancer cells after organoid dissociation were orthotopically injected into the pancreas tail of syngeneic immunocompetent C57Bl/6J or B6129SF1/J animals (*n* = 41 mice). Mouse cell cultures for implantation were sex matched as well as matched to the genetic background of the host animal to avoid graft rejection and immunogenicity^71^. All mice were between 2 and 5 months old at the time of the experiment, purchased from Charles River Laboratories or bred in house (some of the F_1_ hybrids). Mice were euthanatized at the humane endpoint and divided into two subtype-specific cohorts (epithelial and mesenchymal) on the basis of their survival at 2.5 weeks (17 and 18 days, except for one mouse that was killed at 16.5 days) for the mice transplanted with mesenchymal organoids and 3.5 weeks (23 and 24 days) for the epithelial transplanted group of mice. All animal experiments were performed in compliance with the European guidelines for the care and use of laboratory animals. The animal study was approved by the Institutional Animal Care and Use Committees (IACUC) of the Technical University of Munich and the local authorities (Regierung von Oberbayern, Munich, Germany; license 55.2-2532.Vet_02-19-174).

#### Orthotopic implantation in nude mice (after in vitro treatments)

In brief, 2,500 epithelial (mouse line ID: 9591) or mesenchymal (mouse line ID: 16992) cells either as control or pretreated with FFX (for 72 h), or 8 Gy irradiation were implanted into the pancreas of 8-week-old, female athymic Crl:NU(NCr)-^F^°^xn1nu^ mice (total *n* = 33, group size *n* = 5–7 as described above for syngeneic implantations). After 2 weeks (14 days) and when the first mouse reached the humane endpoint, the entire cohort (for both epithelial and mesenchymal) was analysed and euthanatized. Mice were purchased from Charles River Laboratories. All mice were kept in dedicated facilities, enriched housing conditions with a 12:12 h light/dark cycle, temperature between 20 and 24 °C, and a relative humidity of 55%. The animal study was approved by the IACUC of the Technical University of Munich and the local authorities (Regierung von Oberbayern, Munich, Germany; license 55.2-2532.Vet_02-18-91).

Animals were subjected to a final anatomical MRI 2 weeks after the cell implantation and subsequently euthanised.

#### MRI tumour volume measurement

For in vivo anatomical imaging, mice were constitutively anaesthetized with isoflurane (1.5–2.5%, O_2_ flow: 2 l min^−1^, CP-Pharma) during the scanning procedure. Animal temperature was maintained between 37–38 °C and breathing was monitored via a respiration pillow sensor (SA Instruments).

All MRI experiments were performed with a small-animal 7 T preclinical scanner (Agilent Discovery MR901 magnet and gradient system, Bruker AVANCE III HD electronics, running ParaVision 7.0.0) with a tuned 1H birdcage resonator (31 mm inner diameter, RAPID Biomedical). T2-weighted anatomical images were acquired with the following parameters: slice number 29, echo time 38 ms, repetition time 6 s, field of view 28 × 24 mm, section thickness 1 mm. Manual segmentations and tumour volume analysis were performed using ITK-SNAP v.3.6.0 (http://itksnap.org).

#### Tissue histology and quantification of metastasis

Tissue was fixed in paraformaldehyde (PFA) for 48 h and embedded in paraffin. Tumours were evaluated on haematoxylin and eosin (H&E)-stained slides and graded as previously described^72^. Organs were evaluated for metastases on multiple (*n* ≥ 5) H&E sections (every section was at least 200 µm from the previous section) per organ. Metastases were validated to be of pancreatic origin using immunohistochemistry by positive nuclear staining for SOX9 (AB5535, EMD Millipore) in all organ metastases and negative nuclear staining for TTF1 (MSK004-05, Zytomed) in lung metastases only.

#### FLASH 3D imaging of PDAC grafts

After fixation for 16 h at 4 °C, in vivo PDAC samples were tissue cleared and immunolabelled using the FLASH protocol^73^ with minor modifications. End-stage PDAC tumours were excised from the mouse and fixed in 4% PFA in phosphate buffered saline (PBS). Cardiac perfusion of the animal with PBS was omitted. To increase tissue permeability and to account for the high stromal density of late-stage PDAC tumours, samples were incubated in dichloromethane at the beginning of the FLASH protocol, as established previously for FLASH staining of non-perfused tissue samples^74^. To this end, samples were dehydrated by stepwise 30-min incubations in increasing concentrations of 30% and 70% methanol (MetOH) in distilled (d)H_2_O, followed by 3 incubations in 100% MetOH. Samples were then incubated in dichloromethane for 3 days, exchanging the chemical to fresh dichloromethane once every day. Samples were washed twice in 100% MetOH for 1 h each and then bleached for 1 day in 15% dimethylsulfoxide (DMSO), 15% H_2_O_2_ in MetOH, followed by a second incubation in fresh bleaching solution overnight. The samples were then rehydrated through 30-min incubations in 75% and 30% MetOH in dH_2_O and washed twice in Dulbecco’s phosphate buffered saline (DPBS) for 1 h. We used FLASH reagent 2 for the antigen retrieval and incubated the samples in 200 mM boric acid, 4 M urea and 8% 3-(decyldimethylammonio)propanesulfonate inner salt (CAS 15163-36-7) in dH_2_O (pH 7) for 1 h at r.t., before increasing the temperature to 54 °C for overnight incubation. The samples were then washed in PBT (0.2% Triton X-100 in DPBS) 3 times for 1 h at room temperature to remove excess reagent 2. Before antibody incubations, samples were blocked in 10% FBS, 1% BSA, 5% DMSO, 0.2% Triton X-100 and 0.02% NaAzide in DPBS for 1 h, before adding antibody dilutions (all at 1:100) in blocking buffer and incubating the samples for 3 days with gentle agitation at r.t. Primary antibodies used were mouse anti-E-cadherin clone 36 (BD biosciences), mouse anti-pan-Keratin clone AE1/AE3 (Cell Signaling), rabbit anti-Vimentin clone D21H3 (Cell Signaling) and rabbit anti-HNF1α/β antibody clone EPR18644 (Abcam). Samples were washed 3 times in DPBS (30 min per wash) and incubated in AlexaFluor-568 conjugated secondary donkey anti-mouse IgG (Invitrogen) antibody (1:1,000), AlexaFluor-647 conjugated secondary donkey anti-rabbit IgG (Invitrogen) antibody (1:1,000) and Hoechst 33342 (1:1,000) in blocking buffer for 3 nights. Samples were washed in DPBS 4 times for 30 min each and stepwise dehydrated through 1-h incubations in 30%, 50%, 75% and twice in 100% methanol in dH_2_O. Samples were immersed in 30%, 70% and twice in 100% methyl salicylate in methanol for 3 h per incubation. After 2 days, the samples were immersed in 2:1 benzyl benzoate and benzyl alcohol. Imaging was carried out on an Andor Benchtop BC43 spinning disk microscope with a ×10 0.45 NA objective (Nikon) and a ×20 0.7 NA objective (Nikon) using 405, 561 and 638 lasers.

Imaris Viewer (9.7.2) was used for gamma correction and 3D visualization of the data sets. We employed Aivia (10.5) for 3D reconstructions, chosen for its advanced pixel classification and 3D object generation capabilities. This was accomplished through manual annotations in the pixel classifier module, with the criteria for annotation being based on signal intensity and localization as described previously^74^. Pan-keratin staining, used in conjunction with Hoechst, enabled the identification of tumour cell strands in PDAC grafts. When these strands were forming tubular structures, annotations commenced from the tube lumen, covering branching structures to elucidate the 3D organization. To circumvent the challenges posed by the dense mass characteristic of PDAC, we strategically omitted segmentation of immediately adjacent cell strands. This approach enabled the creation of 3D visual representations where growth patterns could be distinctly observed and compared to one another.

#### 2D cell culture of murine and human PDAC cells

We collected primary tumour cells from various genetically engineered mouse models. The 2D cell cultures of murine epithelial and mesenchymal cells were maintained in a humified atmosphere at 37 °C and 5% CO_2_. Cells were cultured in 75 cm^2^ flasks (Thermo Fisher) or 10 cm^2^ culture dishes (Corning) in Dulbecco’s modified Eagle’s medium (DMEM) high glucose with 10% (v/v) fetal bovine serum (FBS) and 1% (v/v) penicillin-streptomycin (P/S) (all from Thermo Fisher). Media changes were performed every 48–72 h. Upon 85% confluency, cells were passaged after washing with cold DPBS and then detached by using 0.05% (v/v) trypsin diluted in DPBS (both from Thermo Fisher). All experiments were performed on cells from between passages 15 and 30. Human 2D cells Panc1, Patu89885S and Patu8988T were cultured in DMEM with 10% FBS and 1% P/S (all from Thermo Fisher), while the human 2D cells DANG, PSN-1, BxPC3 and MiaPaCa2 were cultured in Roswell Park Memorial Institute (RPMI) 1640 medium (Thermo Fisher) with 10% FBS and 1% P/S.

#### 3D PDAC murine culture in floating collagen gels

PDAC floating collagen gels were generated as previously described^14^. Briefly, after washing the cells with cold DPBS, they were trypsinized for 5 min, counted in a Neubauer Chamber and after 3 series of dilutions (first with a concentration of 50,000 cells per ml, second with 2,500 cells per ml, and third with 500 cells per ml), 20 cells per gel were used as a final concentration. Carefully, in a 50 ml conical falcon tube (Corning), the following were mixed vigorously in the following order: media, cell suspension, 10 + 1 neutralizing solution (550 nM HEPES (Sigma-Aldrich) in 11× PBS (Biochrome), house made, pH adjusted to 7.4 with NaOH) and Collagen Type I from rat tail (Corning), and 400 µl of the mixture was distributed in a 24-well plate or 200 µl in a 48-well plate (both from Corning). The final collagen concentration was kept stable at 1.3 mg ml^−1^ unless stated otherwise (Extended Data Fig. 1a,b). Cell culture plates were immediately transferred to a cell culture incubator at 37 °C and 5% CO_2_ and left for 1 h to polymerize. Afterwards, 600 µl (24-well plate) or 300 µl (48-well plate) of the respective media was added and the gels carefully detached by encircling them with a 10 µl tip. Media changes were performed initially after 72 h and then every 48 h.

#### Analysis of extreme limiting dilution

The extreme limiting dilution analysis (ELDA) was performed as described previously^75^. Briefly, after a series of dilutions as mentioned above, we used the last dilution of 500 cells per ml to seed 4 different densities: 20 cells per gel, 10 cells per gel, 3 cells per gel and 1 cell per gel. For every cell density, at least 8 gels were tested and after 13 days of organoids culture, the positive reactions were counted (gels containing at least 1 organoid). Then, using the publicly available software from the Walter and Eliza Hall Institute of Medical Research^76^, we plotted the logarithmic non-responding fraction to cell dose and estimated the potency of our cells to form multicellular branched organoids (B-SFUs). For the secondary and tertiary analysis of epithelial and mesenchymal organoids, we followed a similar strategy as described previously^14^.

#### 3D human PDO culture in Matrigel

Patient-derived organoids were first isolated as previously described^47^, and cultured in 50 µl Matrigel domes in 24-well plates. For organoid passaging upon confluency, we incubated them for 5 min on ice with 500 µl Cell Recovery Solution (Corning). The domes were then mechanically disrupted by adding 1 ml of cold DPBS and mechanically scrubbing the domes. After collection in Falcon tubes, the cultured organoids were left on ice for a 30-min incubation, followed by an initial centrifugation step at 500 *g* for 5 min at 4 °C, mechanical disruption of the pellet (with a 1,000 µl tip) to remove remaining Matrigel and another centrifugation step leading to the final organoid pellet. This was finally resuspended in the required amount of GFR Matrigel (Corning) and plated on 24-well plates. After solidifying, media were added for the establishment and expansion of PDOs (full PDO media).

#### 3D human PDO culture in floating collagen gels

To culture PDOs in floating collagen gels, we repeated the procedure of splitting the PDOs with the addition of a trypsinization step after the first centrifugation. After cell counting, 10,000 cells per gel were seeded in 24-well plates for continuous cell culture. To exclude cell density bias in the morphological comparison between Matrigel- and collagen-grown PDOs, we seeded after trypsinization 10,000 cells per floating collagen gel and 10,000 cells per Matrigel dome. The mixture for the floating collagen gels consisted of cells in suspension, DMEM high glucose with P/S and primocin, neutralizing solution and Collagen Type I (all identical as mentioned in the section above). For the continuous cell culture of PDOs in floating collagen gels, media changes were performed on Days 4, 7 and 10, and at Day 13, the PDOs were passaged in fresh collagen gels. At Day 13 (or upon confluency), we collected the collagen gels in 50 ml Falcon tubes and performed a 15-min enzymatic digestion at 37 °C using 1.5 mg ml^−1^ Collagenase Type 4 (Worthington) diluted in DMEM high glucose with P/S and primocin. Cold DPBS was added to the mixture and the mixture centrifuged at 500 *g* for 5 min. The cell pellet was trypsinized for 5 min at 37 °C and then trypsin was quenched with 250 µg ml^−1^ Soybean Trypsin Inhibitor (STI, Thermo Fisher). Afterwards, 10,000 cells were seeded into collagen gels (as described above). For all functional experiments performed in PDOs (media component screening, imaging, RNA sequencing), a final concentration of 2,000 cells per gel was used unless stated otherwise.

#### Full PDO media

For the establishment, expansion and continuous cell culture of PDO, the following media composition was used: DMEM/F-12 (Thermo Fisher), 5 mg ml^−1^ d-glucose (Sigma-Aldrich), 0.5% ITS premix (Corning), 5% Nu-Serum (Corning), 1× P/S (Thermo Fisher), 25 μg ml^−1^ bovine pituitary extract (Thermo Fisher), 100 ng ml^−1^ cholera toxin (Sigma-Aldrich), 1 µM dexamethasone (Sigma-Aldrich), 10 mM nicotinamide (Sigma-Aldrich), 100 µg ml^−1^ primocin (Invivogen), 5 nM 3,3,5-triiodo-l-thyronine, 0.5 µM A83-01 (Stemcell), 10% R-spondin conditioned media (the HEK293T R-spondin-1 overexpressing cell line was provided by the Hubrecht Institute (Utrecht, the Netherlands)), 100 ng ml^−1^ Wnt3a (R&D Systems) and 10 µM Y-27632 (only upon seeding, Biomol). All components can be found in the Supplementary Table 1.

#### Base PDO media

A reduced version of the full PDO media was designed to allow the breaking of symmetry and possibly the formation of first branching events. The following components were removed from the full PDO media to form the base PDO media: 25 μg ml^−1^ bovine pituitary extract, 100 ng ml^−1^ cholera toxin, 0.5 µM A83-01, 10% R-spondin conditioned media and 100 ng ml^−1^ Wnt3a. All components of the base PDO media can be found in Supplementary Table 2.

#### Basal branching PDO media

Using the base PDO media as our basis, we further enhanced the media by the timely addition of growth factors and inhibitors. The following components were used: 50 ng hEGF (Days 0–13 of culture, 236-EG, R&D Systems), 10 ng hHGF (Days 0–9 of culture, 294-HG, R&D Systems), 25 ng hRspondin-1 (Days 0–7 of culture, 4645-RS, R&D Systems), 50 ng hNoggin (Days 0–13 of culture, 6057-NG/CF, R&D Systems), 50 ng of hFGF-10 (Days 0–13 of culture, 100-26, Peprotech), 3 µM ROCK inhibitor Y-27632 (Days 0–3 of culture, 10005583, Biomol), 5 µM iCRT14 (Days 0–7 of culture, 4299, Tocris), 1% B27 supplement (Days 0–13 of culture, 17504044, Thermo Fisher) and 500 µM *N*-acetyl-cysteine (NAC) (Days 0–13 of culture, A0737, Sigma-Aldrich). All components of the basal branching PDO media can be found in Supplementary Table 3.

#### Chemical perturbations

To identify novel pathways involved in the PDAC branching organoids morphogenesis, chemical perturbations using the following growth factors and inhibitors were performed: 10 µM AG1478 (EGFR inhibitor, Sigma-Aldrich), 5 ng mEGF (R&D Systems), 100 ng ml^−1^ Wnt3a (R&D Systems), 5 µM XAV939 (potent tankyrase (TNKS) inhibitor, 3748, Tocris), 5 µM iCRT14 (potent inhibitor of β-catenin transcription (CRT), 4299, Tocris), 5 ng mHGF (2207-HG, R&D Systems), 10 µM DAPT (g-secretase inhibitor, 2634, Tocris), 10 µM GANT61 (GLI antagonist, 3191, Tocris), 100 ng ml^−1^ h/m-Ihh (1705-HH, R&D Systems), 100 ng ml^−1^ h/m-Shh (1845-SH, R&D Systems), 2 µM Sant-1 (potent, cell-permeable inhibitor of Sonic hedgehog signalling, 1974, Tocris), 5 ng TGFβ-1 (100-21, Peprotech), 5 µM A83-01 (potent ALK inhibitor, including ALK5, 72022, Stemcell) and 1× StemXVivo EMT-inducing media supplement (CCM017, R&D Systems).

For the FOLFIRINOX experiments (Fig. 6), fluorouracil (Medac), irinotecan hydrochloride (Fresenius Kabi) and oxaliplatin (Fresenius Kabi) were generously provided by the Pharmacy at Klinikum rechts der Isar, TUM.

For the anticancer library 3D validation experiments (Fig. 7 and Extended Data Fig. 6), the following drugs were purchased from Selleckchem: Trametinib GSK 1120212 (S2673), KU60019 (S1570), Poziotinib (S7358), AZD5153 6-hydroxy-2naphthoic acid (S8344), ML264 (S8196), Birinapant TL32711 (S7015), R05126766 CH5126766 (S7170), SF1670 (S7310), JIB-04 (S7281), Adavosertib MK-1775 (S1525), Abexinostat PCI-24781 (S1090), Saracatinib AZD0530 (S1006) and Onametostat JNJ-64619178 (S8624). All drugs were diluted in DMSO according to manufacturer instructions.

#### Anticancer 2D library screening

The cherry-picked drug library consisting of 100 inhibitors targeting various relevant cancer pathways was purchased from Selleckchem (L2000-Z424793-100 µl-1 mg). The two SUMO inhibitors ML-93 and TAK-981 were from Millennium Pharmaceuticals/Takeda and later added to the library. The APE1/Ref-1 redox-specific inhibitor APX2009 (ref. ^77^) was a kind gift from Mark R. Kelley (Indiana University, Indiana, USA). Except for A-1210477 (2 mM) and APX2009 (100 mM), the starting maximum stock concentration of all drugs was 10 mM. Serial 7-point 3-fold dilutions of the drugs were prepared in DMSO, pipetted into 384-well plates and stored at −80 °C. Cells were seeded out in white 96-well plates at a density of 1,000 cells (mesenchymal) and 2,000 cells (epithelial) per well in 100 µl DMEM growth medium. The following day, the drug library was transferred from the drug plates to the cells at a 1:1,000 dilution (0.1 µl per well) using a 96-pin replicator pin tool (V&P Scientific). Each drug was analysed in technical duplicates. After each transfer step, the pins were cleaned using DMSO/ethanol (1:1), dried on blot paper for 15 s and then cleaned in isopropanol twice. Cell viability was measured with CellTiter-Glo reagent (Promega) after 72 h of treatment. Therefore, the plates were adjusted to room temperature for ~30 min and then 25 µl of CellTiter-Glo was added to each well. After gentle shaking and a 15-min incubation, luminescence was measured on a FLUOstar OPTIMA microplate reader (BMG Labtech). The AUC and the half-maximal growth inhibitory concentration (GI_50_) values were calculated from the results using the RStudio software tool with a script based on the GRmetrics methodology^78^.

#### Irradiation of 2D murine cells

All irradiation experiments were performed using the Gulmay RS225A irradiation device (Gulmay Medical). Radiation was delivered at 200 kV and 15 mA with a dose rate of 0.90 Gy min^−1^ using a copper filter and table setting at 500 mm.

#### Brightfield imaging

For brightfield microscopy, organoids were imaged with a Leica DM IL LED microscope (Leica) equipped with a DFC 450C or DMC4500 digital camera at ×5, ×10 and ×20 magnifications.

#### Image and data analysis

Images were analysed using ImageJ 1.53c^79^ or GIMP 2.10. The 3D image reconstruction of phalloidin/DAPI was performed using Imaris (8.2.0, Oxford Instruments). Numerical data were analysed and the graphs made using Graphpad Prism (v.9.0.2 and 10.1.0). For figure generation, we used Inkscape (v.1.2.1). Figures 1a, 3g, 5d, 6a and 7c, and Extended Data Figs. 4e, 7b and 9a were created with BioRender.com.

#### Structural characterization of the organoids

The structural characterization and quantification of the organoids was done using ImageJ 1.53c (v.2.6.0). First, the pixel size of the images was adjusted to the corresponding known distance in μm. Then, the major axis length and the thickness of the core branch were measured using the ‘straight’ tool. The lumen size (μm^2^) was the sum area of the lumens present, whose area was determined using the ‘polygon’ tool and then measured with the ‘measure’ function. The numbers of main branches, nodes, spiky branches, lumens and terminal end buds were manually quantified. The granularity level was qualitatively evaluated. The area of the core of the star-like organoids was quantified as mentioned previously using the ‘polygon’ tool, and the perfect circle area was calculated by measuring the diameter of the core using the ‘straight’ tool.

#### Manual analysis of morphological clustering

For the generation of a phenotypic organoid PDAC landscape, a large collection of 4,113 control organoids (untreated) was used, originally from 6 (3 epithelial and 3 mesenchymal) primary murine PDAC lines.

For the epithelial subtype derived from the line ID: 9591, 4 major categories were recognized on the basis of their morphological features and 5 subcategories. The TEBBO family of organoids develops as a main trunk from which multiple sub-branches arise, forming the characteristic terminal end buds, with a seamless lumen connecting the entire organoid. According to their size, TEBBOs can be either TEBBO-large or TEBBO-small. If TEBBOs are present with thinner branches and no continuous hollow lumen, organoids are clustered as TEBBO-immature. If any organoid appears relatively more granular or have a swollen lumen, then it is categorized as a TEBBO-like organoid. The second major category are the thick branched organoids with thick branches, lumen sites and irregular distribution of branches. Three subcategories were identified: the classical thick branched, the granular thick branched (with granular appearance of cells) and the granular/cystic with granular appearance and swollen lumens. Next category is the cystic branched organoids with pronounced lumen swelling. The last epithelial category is tree-like organoids, with thin, mostly single-cell layered branches largely spread in an extended area and without an obvious formation of a lumen.

For the mesenchymal subtype derived from the line ID: 16992, 3 major categories were recognized on the basis of their morphological features and 5 subcategories. The branched mesenchymal organoids exhibit a strong, thick organoid core with multiple invasive branches. If the main branches appear thinner, the organoids were classified as branched mesenchymal-thin organoids. Star-family organoids initially grow as a clump of cells which, during development, reaches a critical mass. Branches then emerge as they break the perfect circular symmetry and invade through the collagen matrix. If the symmetry is not broken, then they remain under the category of clump (filled lumen, not cystic). Depending on the length and thickness of the branches, organoids can be categorized either as star-like or star-like branched if the branches appear longer than the main core (clump) parts. The majority of the mesenchymal categories consist of the firework-family organoids which, unlike their similar star-like organoids, do not exhibit a perfectly solid symmetric organoid core but rather multilayer thick invasive branches forming a continuous net-like structure. If the main core is thin and the individual branches are visible, then it is categorized as firework-thin and in case it consists only of a few long main branches, then we clustered them as firework-branched. Similar analyses were performed for the other mouse lines: E-line IDs: 8442, 53631 and M-line IDs: 8028, 9091. Control (untreated) organoid images of the E-line ID: 9591 from our previous publication^14^ were also included for the morphological analysis (to increase the total organoid numbers).

#### Manual morphological-clustering analysis under treatment

Under the influence of various treatment approaches from chemotherapy (FOLFIRINOX) and irradiation up to treatment with drugs from the anticancer library, new morphological clones arose and were categorized. Due to lower number of organoids analysed and to simplify the analysis of the TEBBO family of organoids, organoids were summarized as TEBBO organoids (including the TEBBO-classical and TEBBO-like organoids). Among the new epithelial categories are the following: thick small organoids, thick-tree, cystic spheres, spiky thick organoids and the scattered phenotype of organoids/cells. In the mesenchymal subtype, the only new emerging phenotype was the scattered organoids/cells.

#### Manual morphological-clustering analysis of PDOs cultured in basal branching PDO media

For the generation of a phenotypic morphological landscape using PDOs, PDOs were cultured inside floating collagen gels in basal branching PDO media which allowed them to undergo branching morphogenesis and exhibit their inherited heterogeneous morphologies. In the PDO line B211, 3 major categories and 2 subcategories were identified, while in the B250 line, 3 major categories and 1 subcategory were identified. Finally, in the line B320, 1 major category with its subcategory was characterized. The lines B211 and B250 share common phenotypes, with the ductal complex phenotype accounting for most organoids analysed. In the ductal complex organoids, sub-branches develop from the main duct and are connect via a lumen which eventually becomes hollow (swells). The ductal stick-like organoids resemble a ductal-like structure with lower complexity compared with the previous category, harbouring a hollow lumen and a minimum number sub-branches (<3). The most aggressive category appears to be the invasive branching spheres which resemble a simplified version of the murine star-like/firework type of organoids, where a central sphere is connected to multiple thin sub-branches invading through the matrix. The branching sphere category, where cells break the perfect symmetry and form the first branching events, was found only in the line B211. A more extreme version of the latter is the multisphere subtype where more than two spheres are interconnected via a ductal site and the hollow lumen. Lastly, the line B320 exhibits similar morphologies to the ductal complex and ductal stick-like categories, except that these do not bear a hollow lumen site and are generally thinner, and are therefore categorized as ductal complex (no lumen) and ductal stick-like (no lumen), respectively.

#### Deep-learning-based clustering of organoid imaging data

For the derivation of imaging-based phenotypes, a deep neural network-based machine learning approach was employed. To obtain a deep representation specific to the microscopic organoid imaging data, a ResNet-50 network was fine-tuned and pretrained on ImageNet for the classification of the six distinct cell lines from which the organoids were grown. The penultimate layer of this network was then subsequently used to extract features specific to the organoids, which were then processed using PCA followed by *t*-SNE. For the training of the network in this pipeline, a data set comprising 4,099 images was used. Figure 3h and Extended Data Fig. 4b show features from a separately kept test set comprising 1,579 images that were not used in neural network training, and were extracted and dimensionality reduced as explained above. To determine the optimal number of clusters, *K*-means clustering with the elbow method was used to identify imaging-based phenotypes, which were visualized in distinct colours. Figures 3j,k and 8k, and Extended Data Fig. 8c,d,k show kernel-density estimates of dimensionality-reduced features of organoids subjected to different treatment options, thus highlighting the effect of the treatment options on visual phenotypic properties.

#### Statistical analysis

For the statistical analysis, we used either Graphpad Prism (v.9.0.2 and 10.1.0) or the R environment for statistical computing (v.4.0.4). No particular statistical method was used to define the sample size and no specific hypothesis was tested. All data were analysed from at least 3 individual experiments unless stated otherwise.

#### Immunostaining

After 13 days of culture (unless indicated otherwise), gels were washed once with DPBS for 10 min, then fixed in 4% (w/v) PFA (Alfa Aesar) for 15 min, followed by a 10-min wash with DPBS and complete quenching of PFA with 0.15 M of Gly diluted in DPBS. Gels were then kept in DPBS at 4 °C for further use. For immunofluorescence staining, cells were permeabilized with 0.2% (v/v) Triton X-100 (Sigma-Aldrich) in DPBS for 10 min at r.t., then washed once with DPBS and incubated overnight at 4 °C with 10% (v/v) normal donkey serum or normal goat serum in 0.1% BSA/DPBS (Carl Roth). After washing with DPBS once, primary antibodies (Supplementary Table 4) were added at the indicated dilutions in 0.1% BSA/DPBS for overnight incubation at 4 °C. This was followed by 3 DPBS washing steps. Gels were then incubated for 3 h at r.t. with secondary antibodies (Supplementary Table 5) diluted in 0.1% BSA/DPBS. Lastly, gels were washed 3 times with DPBS, incubated for 2 min with DAPI, followed by 3 washes with DPBS and 3 washes with dH_2_O before mounting on slides with aqua-Poly/Mount (Polysciences).

#### In vitro hypoxia staining

Briefly, organoids were cultured as described above for 13 days. On Day 13, gels were incubated for 3 h with 10 µM Image-iT green hypoxia reagent (Thermo Fisher). Organoids were carefully washed, fixed (as described above) and then stained with DAPI solution for 5 min at r.t. (without permeabilization). Afterwards, the organoids were imaged using confocal microscopy.

#### Immunofluorescence confocal imaging

For the acquisition of immunostaining images, we used a laser scanning confocal microscope (Olympus FluoView 1200, Olympus) equipped with the following objectives: Olympus UPlanSAPO ×60 1.35, Olympus UPlanSAPO ×40 1.25 solid immersion lens oil immersion objectives, Olympus UPlanSAPO ×20 0.75 and Olympus UPlanSAPO ×10 0.40 air immersion objectives (Olympus). For the 3D maximum projection (Extended Data Fig. 3a), organoids were imaged using either the confocal laser scanning microscope LSM 880 with Airyscan module (Carl Zeiss) (objective ×10 0.4 NA EC Plan-Neoflura (Zeiss), lasers 405 and 633 nm), or the confocal laser scanning LSM 980 (Carl Zeiss) (objective ×10 0.45 NA W C-apochromat (Zeiss), and lasers 405 and 639 nm).

#### Seahorse experiment

Mitochondrial respiration and glycolysis were measured with the Seahorse XFe 96 Analyzer (Agilent Technologies). To achieve this, 20,000 cells from individual organoid phenotypes per well were plated in Seahorse 96-well cell plates. After overnight incubation of the cells, oxygen consumption rate and extracellular acidification rate (ECAR) were measured by the injection of 2 μM oligomycin (Sigma-Aldrich), 1 μM FCCP (Sigma-Aldrich) and 1 μM antimycin A (Sigma-Aldrich), together with 1 μM rotenone (Sigma-Aldrich). Glycolysis was measured by additional injection of 100 mM 2-deoxy-d-glucose (Sigma-Aldrich). The OCR and ECAR results were normalized to the fluorescence intensity of 10 µM Hoechst (Thermo Fisher) to stain and quantify DNA content, which reflects live cell account.

#### RNA isolation

The 2D cells were washed once with cold DPBS, collected with cell scrapers in RLT Plus buffer with β-mercaptoethanol, pressed through 1 ml Sub-Q syringes (BD) and then stored at −80 °C until RNA isolation. For the 3D collagen organoids, a Collagenase Type V (Worthington) digestion was first performed for 12–15 min until the collagen matrix was no longer visible. Then the organoids were washed once with cold DPBS, the organoid pellet was collected in RLT Plus buffer with β-mercaptoethanol, pressed through 1 ml Sub-Q syringes (BD) and then stored at −80 °C until RNA isolation. The cell/organoid lysates were first homogenized for 2 min at maximum speed using QIAshredders (Qiagen). The total RNA was then isolated using the RNeasy Plus micro kit (Qiagen) according to manufacturer instructions, with an additional step of silica column DNA digestion for 15 min using the RNase-Free DNase set (Qiagen) to ensure the highest RNA purity.

#### Bulk RNA-sequencing

Poly(A)-RNA library preparation for bulk sequencing was performed as described previously^80^. First, RNA was reverse transcribed using Maxima RT polymerase with an oligo-dT primer containing barcodes, unique molecular identifiers (UMIs) and an adaptor (Thermo Fisher). The complementary (c)DNA from each sample was barcoded. Using a template switch oligo (TSO), the cDNA 5’-ends were extended, and the full-length cDNA amplified with the primer binding to the TSO-site and the adaptor. To fragment the cDNA, the NEB UltraII FS kit was used. Next, end repair and A-tailing was performed, a TruSeq adapter was ligated and the 3’-end fragments were amplified by primers with Illumina P5 and P7 overhangs. Compared to previously published studies^80^, the P5 and P7 sites were exchanged to facilitate sequencing of cDNA in read1 and the barcodes and UMIs in read2 to obtain an optimal cluster recognition. Finally, the NextSeq 500 system (Illumina) was used to sequence the library with 65 cycles for the cDNA (read1) and 18 cycles for the barcodes and UMIs (read2). For data processing, the previously published Drop-seq pipeline (v.1.0) was used to generate sample- and gene-wise UMI tables^81^. For alignment, the reference genome (GRCm38) for mouse cells/organoids or GRCh38 for PDOs was used. Transcript and gene definitions were used according to GENCODE v.M25 for mouse and GENCODE v.38 for PDOs.

#### Bulk RNA-sequencing analysis

We used the R environment for statistical computing (v.4.0.4) to perform high-throughput gene-expression analysis for the conditions mentioned in the main text^82^.

#### Differential gene-expression analysis

To screen for differentially expressed genes between experimental conditions, we performed a genome-wide differential gene-expression analysis for RNA-seq count data using a negative binomial generalized linear model as implemented in the DESeq2 R package^83^. We used the following parameters to calculate dispersion estimates: cell line ID, culture dimension (2D, 3D), culture media conditions (for the PDOs), cell origin (epithelial, mesenchymal), organoid morphology (bulk, clonal populations) and treatment. A false discovery rate (FDR) < 0.1 was considered significant for the individual comparisons.

#### Gene-set-enrichment analysis

We used the fgsea R package^84^, and Wald statistics as the gene-level statistics, to subject individual differential gene-expression signatures between two conditions to gene set enrichment analysis (GSEA). Gene sets were retrieved from MSigDb (v.7.3)^85,86^. We illustrated certain pathways from the enrichment results using custom R code. For specific pathways, we illustrated specific leading-edge genes between compared conditions after *Z*-score transformation (all rows scaled to have a mean of 0 and a variance of 1) using the pheatmap R package^87^. EMT scores were derived from epithelial and mesenchymal organoids using single-sample GSEA as implemented in the aREA function of the viper R package^88^.

#### Single-cell RNA-sequencing library preparation and sequencing

Cells from selected 2D cell lines (IDs: 9591, 16992) were counted, diluted to an appropriate cell number in ice-cold DPBS and loaded on a 10× Chromium Next GEM Chip G to generate gel beads in emulsion (GEMs). Single-cell GEM generation, barcoding and library construction were performed using 10× Chromium Single Cell 3’ v.3.1 chemistry according to manufacturer instructions. Quality and library size of resulting cDNA and generated libraries were determined on an Agilent Bioanalyzer 2100 system using the HS DNA kit (Agilent). The library was sequenced on a NextSeq 500 system (Illumina), with 26 cycles for the barcodes and UMI in read1 and 58 cycles for the cDNA in read2.

### Single-cell RNA-sequencing analysis

#### Raw data processing

Raw sequencing reads from 9591-2D and 16992-2D models were processed using CellRanger v.6.1.2. Reads were aligned to GRCm39 (mm39) from the Ensemble 105 release. After filtering out empty droplets in CellRanger, 17,412 cells from sample 9591-2D and 9,257 cells from sample 16992-2D remained for further analysis.

#### Single-cell data processing

The single-cell count data were processed using Scanpy (v.1.9.1)^89^ following a published best-practice workflow^90^.

#### Quality control

The joint distribution of count depth per cell, the number of expressed genes per cell and mitochondrial read fraction per cell was considered to filter low-quality cells. This filtering was performed for the 9591-2D sample using the following thresholds: cells with >27,500 counts, <250 expressed genes and 20% or more reads aligned to mitochondrial genes were filtered out. In addition, genes detected in <20 of cells were removed. The resulting data set for 9591-2D consists of 16,747 cells and 11,831 genes. For 16992-2D, the following thresholds were applied: cells with >32,500 counts, <390 expressed genes and 20% or more reads aligned to mitochondrial genes were filtered out. In addition, genes detected in <20 of cells were removed. The resulting data set for 16992-2D consists of 9,190 cells and 11,754 genes.

#### Highly variable gene selection and dimensionality reduction

Upon quality control, the data were normalized, log+1 transformed, and highly variable genes (HVG) were selected. Specifically, the pooling method from scran (1.24.0)^91^, calculateSumFactors() (min.mean=0.1), was applied for normalization (min-mean=0.1), followed by log+1 transformation using the Scanpy function ‘sc.pp.log1p’. For HVG selection, 4,000 HVGs per organoid were selected using the Scanpy preprocessing method ‘sc.pp.highly_variable_genes with flavour = ‘cell_ranger’’.

These HVGs were used to generate a uniform manifold approximation and projection (UMAP)^92^ embedding to visualize the data in 2D. The UMAP embedding was generated from a *k*-nearest neighbour (kNN) graph (with *k* = 15 using Scanpy function ‘sc.pp.neighbours’) built using Euclidean distances on the principal component space (PC space, 50 principal components) from Scanpy preprocessing ‘sc.pp.pca’.

#### Clustering and annotation

To cluster gene-expression data, graph-based Louvain clustering was applied to the kNN graph. The Scanpy tool ‘scanpy.tl.louvain’ was used at a resolution of 0.55 for the 9591-2D organoid and at a resolution of 0.28 for the 16992-2D organoid. This resulted in 5 clusters for each data set. Marker genes for each cluster were computed using a Welch *t*-test with false discovery rate correction using the Benjamini–Hochberg method. The top marker genes were used to annotate the clusters.

#### Enrichment analysis of gene sets within clusters

To interpret clusters, we performed enrichment analysis of hallmark gene sets^85^ on the cluster’s marker genes using fgsea (v.1.20.0)^84^. The gene sets corresponding to the EMT were retrieved from the hallmark gene set collection of MSigDB^85^, as well as the S1 and S2 EMT gene sets in ref. ^26^ using gmtPathways. Afterwards, all genes in the gene sets were mapped to *Mus musculus* using a mapping from http://www.ensembl.org. Gene set activity was measured using AUCell (v.1.16.0)^93^. Using ‘AUCell_buildRankings’ and ‘AUCell_calcAUC’ in AUCell v.1.16.0, all genes in the clusters were ranked on the basis of their differential expression, and an AUC score of the gene sets was calculated.

#### Derivation of organoid phenotype signatures

Signatures associated with each organoid phenotype were derived by identifying genes with higher average expression relative to the rest of the models. Raw RNA-seq counts from all organoid phenotypes (*n* = 3 per phenotype) were first modelled in DESeq2 with the design ‘~ 0 + Phenotype’. For each phenotype, we performed differential expression, contrasting against all other phenotypes. Signature genes were defined as those with a Benjamini–Hochberg-adjusted *P* < 0.05 and a log_2_ fold change >2. To avoid signatures simply reflecting epithelial or mesenchymal properties, genes occurring in more than one phenotype were removed from signatures.

#### Signature scoring of human PDAC

ScRNA-seq from ref. ^37^ was previously accessed using malignant cells that were identified on the basis of automated annotation with SingleR^94^ and inference of genomic copy number alterations^26^. Cells were scored for each of the organoid signatures using UCell (v.2.4.0)^95^, first converting mouse genes from the signatures to human orthologues. To evaluate the distribution of signature activity across malignant phenotypes, we integrated cells from each patient sample using Harmony (v.1.0.3)^96^ through the HarmonyIntegration method provided by Seurat (v.5.0.0)^97^.

### Reporting summary

Further information on research design is available in the Nature Portfolio Reporting Summary linked to this article.

## Supplementary information

Main Supplementary Information Supplementary tables.

Reporting Summary

Supplementary Data Information on targeted drug and FFY treatments, on PDO-line clinical data, on the implantation of the different organoid phenotypes and on implantation after cell treatment with FFX and 8-Gy irradiation.

## Source data

Source Data Figs. 1 and 3–8 and Extended Data Figs. 1–6 and 8 Source data.

## Data Availability

The bulk RNA-sequencing data are available from the GEO database under accession code GSE261159. The single-cell RNA sequencing data are available in Zenodo^98^. All relevant data supporting the findings of this study are available within the paper and its Supplementary Information. Source data for the figures are provided with this paper.

## Code availability

The code of MOrPHeMap is available in Zenodo^99^. Single-cell RNA-seq codes are available in GitHub^100^.
