# Supplementary material for: Heterogeneity-driven phenotypic plasticity and treatment response in branched-organoid models of pancreatic ductal adenocarcinoma
Source: Nat Biomed Eng. 2024 Dec 10;9(6):836–64. doi: 10.1038/s41551-024-01273-9 (PMC12176653; doi:10.1038/s41551-024-01273-9)
Supplement: Supplementary file 1 — Supplementary tables. [file 41551_2024_1273_MOESM1_ESM.pdf]

nature biomedical engineering

Supplementary information https://doi.org/10.1038/s41551-024-01273-9

Heterogeneity-driven phenotypic plasticity and treatment response in branched-organoid models of pancreatic ductal adenocarcinoma

In the format provided by the authors and unedited

Supplementary Table 1. Full PDO Media composition including components, final concentrations, manufacturer and catalogue numbers.

Full PDO Media	Final Concentration	Manufacturer	Cat. Number
DMEM-F12	NA	ThermoFisher Scientific	31330-038
D-Glucose	5 mg/mL	Sigma Aldrich	G8270
ITS premix	0.5%	Corning	354350
Dexamethasone	1 μM	Sigma Aldrich	D1756
Cholera toxin	100 ng/mL	Sigma Aldrich	C8052
Penicillin/Streptomycin	1x	ThermoFisher Scientific	15140-122
Nu Serum	5%	Corning	355500
Bovine Pituitary Extract	25 μg/mL	ThermoFisher Scientific	13028-014
Primocin	100 μg/mL	InvivoGen	Ant-pm-1
A83-01	0.5 μM	Stemcell	72022
Nicotinamide	10 mM	Sigma Aldrich	N3376
3,3,5-Triiodo-L-thyronine	5 nM	Sigma Aldrich	T2877
Wnt3a	100 ng/mL	R&D Systems	5036-WN-500
Y-27632	10 μM	Biomol	10005583

Afterwards, in the Full PDO Media is added at a final concentration 10% the R-spondin conditioned media.

Y-27632 is only added after passaging (Day 0-3).

Supplementary Table 2. Base PDO Media composition including components, final concentrations, manufacturer and catalogue numbers.

Base PDO Media	Final Concentration	Manufacturer	Cat. Number
DMEM-F12	NA	ThermoFisher Scientific	31330-038
D-Glucose	5 mg/mL	Sigma Aldrich	G8270
ITS premix	0.5%	Corning	354350
Dexamethasone	1 μM	Sigma Aldrich	D1756
Penicillin/Streptomycin	1x	ThermoFisher Scientific	15140-122
Nu Serum	5%	Corning	355500

Primocin	100 μg/mL	InvivoGen	Ant-pm-1
Nicotinamide	10 mM	Sigma Aldrich	N3376
3,3,5-Triiodo-L-thyronine	5 nM	Sigma Aldrich	T2877
Y-27632	3 μM	Biomol	10005583

Y-27632 is only added after passaging (Day 0-3).

Supplementary Table 3. Basal Branching PDO Media composition including components, final concentrations and timepoints of treatment.

Basal Branching PDO Media	Final Concentration	Time-point treatment (days)
hEGF	50 ng	0-13
hHGF	10 ng	0-9
hRspondin-1	25 ng	0-7
hNoggin	50 ng	0-13
hFGF-10	50 ng	0-13
iCRT14	5 μM	0-7
B27	1 %	0-13
NAC	500 μM	0-13
Y-27632	3 μM	0-3

Supplementary Table 4. Primary Antibodies including information on clones, conjugation, host, catalogue number, supplier and dilution.

Epitope [Clone]	Conjugation	Host	Catalogue number	Supplier	Dilution
Phalloidin	Atto-647		65906	Sigma	1:250
E-cadherin	Alexa-488	rabbit mAb (24E10)	3199	Cell Signaling	1:50
E-cadherin	Alexa-488	mouse mAb (Clone36/RUO)	560061	BD Biosciences	1:50
N-cadherin	-	mouse mAb (13A9)	14215	Cell Signaling	1:100

Ki67	-	rabbit pAb	ab15580	Abcam	1:300
Ki67	-	rat mAb SolA15	14-5698-82	Thermo Fisher Scientific	1:100
Zeb1 (H-102)	-	rabbit pAb	Sc-25388	Santa Cruz	1:100
Yap	-	rabbit pAb	4912S	Cell Signalling	1:100
ZO-1	Alexa-594	mouse mAb	339194	Invitrogen	1:100
Vimentin (V9)	-	mouse mAb	MAB3578	Abnova	1:100
Vimentin (D21H3)	-	rabbit mAb	5741	Cell Signaling	1:100
beta-catenin	-	mouse mAb	610153	BD	1:150

Supplementary Table 5. Secondary Antibodies including information on host, species reactivity, conjugation, catalogue number, supplier and dilution.

Host	Species reactivity	Conjugation	Catalogue number	Supplier	Dilution
Goat	Rabbit	Alexa Fluor Plus 555	A32732	Thermo Fisher Scientific	1:250
Donkey	Rabbit	Alexa Fluor 546	A10040	Thermo Fisher Scientific	1:250
Goat	Mouse	Alexa Fluor 546	A11030	Thermo Fisher Scientific	1:250
Goat	Rabbit	Alexa Fluor 488	A11034	Thermo Fisher Scientific	1:250
Goat	Rat	Alexa Fluor 594	A11007	Thermo Fisher Scientific	1:250
