# Supplementary material for: Heterogeneity-driven phenotypic plasticity and treatment response in branched-organoid models of pancreatic ductal adenocarcinoma
Source: Nat Biomed Eng. 2024 Dec 10;9(6):836–64. doi: 10.1038/s41551-024-01273-9 (PMC12176653; doi:10.1038/s41551-024-01273-9)
Supplement: Supplementary file 2 — Reporting Summary [file 41551_2024_1273_MOESM2_ESM.pdf]

nature portfolio

Corresponding author(s): Maximilian Reichert

Last updated by author(s): Apr 10, 2024

Reporting Summary

Nature Portfolio wishes to improve the reproducibility of the work that we publish. This form provides structure for consistency and transparency in reporting. For further information on Nature Portfolio policies, see our Editorial Policies and the Editorial Policy Checklist.

Statistics

For all statistical analyses, confirm that the following items are present in the figure legend, table legend, main text, or Methods section.

n/a	Confirmed	
☐	☒	The exact sample size (*n*) for each experimental group/condition, given as a discrete number and unit of measurement
☐	☒	A statement on whether measurements were taken from distinct samples or whether the same sample was measured repeatedly
☐	☒	The statistical test(s) used AND whether they are one- or two-sided<br>*Only common tests should be described solely by name; describe more complex techniques in the Methods section.*
☐	☒	A description of all covariates tested
☒	☐	A description of any assumptions or corrections, such as tests of normality and adjustment for multiple comparisons
☐	☒	A full description of the statistical parameters including central tendency (e.g. means) or other basic estimates (e.g. regression coefficient) AND variation (e.g. standard deviation) or associated estimates of uncertainty (e.g. confidence intervals)
☐	☒	For null hypothesis testing, the test statistic (e.g. *F*, *t*, *r*) with confidence intervals, effect sizes, degrees of freedom and *P* value noted<br>*Give P values as exact values whenever suitable.*
☒	☐	For Bayesian analysis, information on the choice of priors and Markov chain Monte Carlo settings
☒	☐	For hierarchical and complex designs, identification of the appropriate level for tests and full reporting of outcomes
☒	☐	Estimates of effect sizes (e.g. Cohen's *d*, Pearson's *r*), indicating how they were calculated

Our web collection on statistics for biologists contains articles on many of the points above.

Software and code

Policy information about availability of computer code

Data collection	Immunostaining images were acquired using a laser scanning confocal microscope (Olympus FluoView 1200; Olympus Corporation). Additional immunofluorescence imaging was performed on an LSM 880 or an LSM 980 confocal laser scanning microscope with the Airyscan module (Carl Zeiss). The libraries of bulk and single cells were sequenced on a NextSeq 500 (Illumina). Brightfield microscopy of organoids was acquired with a Leica DM IL LED microscope (Leica, Wetzlar, Germany). MRI was performed with a small animal 7 T preclinical scanner (Agilent Discovery MR901 magnet and gradient system, Bruker AVANCE III HD electronics, running ParaVision 7.0.0). FLASH was imaged on Andor Benchtop BC43 spinning-disk microscope. Mitochondrial respiration and glycolysis were measured with the Seahorse XFe 96 Analyzer (Agilent Technologies). For the drug treatments, luminescence was measured on a FLUOstar OPTIMA microplate reader (BMG Labtech, Ortenberg, Germany).
Data analysis	Images were analysed using ImageJ 1.53c or GIMP 2.10. Three-dimensional image reconstructions were performed using Imaris (8.2.0 or 9.7.2 Viewer, Oxford Instruments). Aivia (10.5) was used for tissue 3D reconstructions. Manual segmentations and tumour-volume analysis from MRI was performed using ITK-SNAP, Version 3.6.0. Numerical data were analysed using Graphpad Prism (ver 9.0.2 and 10.1.0). The graphs were made using Graphpad. For the statistical analysis we used either Gaphpad Prism (ver 9.0.2 and 10.1.0) or the R environment for statistical computing (v4.0.4). The single-cell-count data was processed using scanpy version 1.9.1. For figure assembling we used Inkscape (ver 1.2.1). Figure 1a, 3g, 5d, 6a, 7c and Extended Data Figures 4e, 7b, 9a were generated with Biorender (BioRender.com).

For manuscripts utilizing custom algorithms or software that are central to the research but not yet described in published literature, software must be made available to editors and reviewers. We strongly encourage code deposition in a community repository (e.g. GitHub). See the Nature Portfolio guidelines for submitting code & software for further information.

Data

Policy information about availability of data

All manuscripts must include a data availability statement. This statement should provide the following information, where applicable:

- Accession codes, unique identifiers, or web links for publicly available datasets
- A description of any restrictions on data availability
- For clinical datasets or third party data, please ensure that the statement adheres to our policy

The bulk RNA-sequencing data are available from the GEO database under accession code GSE261159. The single-cell RNA sequencing data are available from the Zenodo repository with the identifier https://zenodo.org/records/10721000. Source data for the figures are available with this paper. All relevant data supporting the findings of this study are available within the paper and its Supplementary Information.

Research involving human participants, their data, or biological material

Policy information about studies with human participants or human data. See also policy information about sex, gender (identity/presentation), and sexual orientation and race, ethnicity and racism.

Reporting on sex and gender	Patients enrolled in the study were not selected based on their sex and gender.
Reporting on race, ethnicity, or other socially relevant groupings	Patients were enrolled in the study solely based on their disease (pancreatic cancer); no information on race, ethnicity or other characteristics was collected or provided for this study.
Population characteristics	Information about the source (surgery or endoscopy), gender, age group (decade), disease stage, treatments, PDAC localization and histological tumour grading at the time of PDO isolation is available as Supplementary Information.
Recruitment	For all patients, a written consent was acquired before sample collection.
Ethics oversight	Experiments involving human material were designed according to the Declaration of Helsinki, and conformed to the Department of Health and Human Services Belmont Report. For the generation of patient-derived organoids, we used either PDO lines previously described (refs. 41,67) or newly generated PDO lines from PDAC patients, upon acquiring their written consent approved by the ethics review board of the Klinikum rechts der Isar der TUM, School of Medicine and Health, Technical University of Munich. Institutional review board (IRB) project-numbers 207/15, 1946/07, 330/19S, 181/17S, 5542/12 and 80/17S.

Note that full information on the approval of the study protocol must also be provided in the manuscript.

Field-specific reporting

Please select the one below that is the best fit for your research. If you are not sure, read the appropriate sections before making your selection.

☒ Life sciences ☐ Behavioural & social sciences ☐ Ecological, evolutionary & environmental sciences

For a reference copy of the document with all sections, see nature.com/documents/nr-reporting-summary-flat.pdf

Life sciences study design

All studies must disclose on these points even when the disclosure is negative.

Sample size	No particular statistical method was used to predetermine sample sizes. The number of biological and technical replicates were choosen on the basis of previous published experimental designs (Randriamanantsoa, Papargyriou et al., 2022). A minimum of 3 biological replicates and 3 technical replicates were used for all experiments, unless stated otherwise (sample sizes for each experiment are stated in the correspoinding figure captions).
Data exclusions	No data were excluded from the analyses.
Replication	Experiments were reliably replicated. Experiments were performed on different days and different batches of organoids (the exact sample sizes of all experiments are stated at the corresponding figure captions). All experiments were analysed from at least 3 individual replicates, unless stated otherwise. The total number of imaged organoids analysed in the study amounted to more than 37,288 (excluding organoids that had been sequenced or used in other assays; for instance, for transplantation models), indicating the robustness of the study.
Randomization	Randomization was not performed.
Blinding	The investigators were not blinded during data collection and analysis.

Reporting for specific materials, systems and methods

We require information from authors about some types of materials, experimental systems and methods used in many studies. Here, indicate whether each material, system or method listed is relevant to your study. If you are not sure if a list item applies to your research, read the appropriate section before selecting a response.

Materials & experimental systems

n/a	Involved in the study
☐	☒ Antibodies
☐	☒ Eukaryotic cell lines
☒	☐ Palaeontology and archaeology
☐	☒ Animals and other organisms
☒	☐ Clinical data
☒	☐ Dual use research of concern
☒	☐ Plants

Methods

n/a	Involved in the study
☒	☐ ChIP-seq
☒	☐ Flow cytometry
☒	☐ MRI-based neuroimaging

Antibodies

Antibodies used

All antibodies are listed in Methods. The Supplementary tables provide information about all antibodies used for organoid stainings, primary antibodies, including information on clones, conjugation, host, catalogue number, supplier/manufacturer and dilution, and secondary antibodies, including information on host, species reactivity, conjugation, catalogue number, supplier/manufacturer and dilution.

Primary antibodies
epitope, conjugation (if applicable), host-clonality, catalog number, manufacturer and dilution.

Phalloidin Atto-647 65906 Sigma 1:250
E-cadherin Alexa-488 rabbit mAb (24E10) 3199 Cell Signaling 1:50
E-cadherin Alexa-488 mouse mAb (Clone36/RUO) 560061 BD Biosciences 1:50
N-cadherin - mouse mAb (13A9) 14215 Cell Signaling 1:100
Ki67 - rabbit pAb ab15580 Abcam 1:300
Ki67 -rat mAb 14-5698-82 Thermo Fisher Scientific 1:100
Zeb1 (H-102) - rabbit pAb Sc-25388 Santa Cruz 1:100
Yap - rabbit pAb 4912S Cell Signalling 1:100
ZO-1 Alexa-594 mouse mAb 339194 Invitrogen 1:100
Vimentin (V9) - mouse mAb MAB3578 Abnova 1:100
Vimentin (D21H3) - rabbit mAb 5741 Cell Signaling 1:100
beta-catenin - mouse mAb 610153 BD 1:150

Additional Ab used for the 3D Tumour stainings
E-cadherin Alexa-488 mouse mAb (Clone36/RUO) 560061 BD Biosciences 1:100
Vimentin (D21H3) - rabbit mAb 5741 Cell Signaling 1:100
Pan-Keratin clone AE1/AE3 -mouse mAb 67306 Cell Signaling 1:100
Hnf1a/b clone EPR18644 -rabbit mAb ab209666 Abcam 1:100

Additional Ab used for IHC (pathology confirmation)
SOX9 -rabbit pAb AB5535 Merck Millipore 1:5000
TTF1 -mouse Ab MSK004-05 Zytomed 1:100

Secondary antibodies
Host-reactivity,conjugation, catalog number, manufacturer and dilution.

Goat anti-Rabbit Alexa Fluor Plus 555 A32732 Thermo Fisher Scientific 1:250
Donkey anti-Rabbit Alexa Fluor 546 A10040 Thermo Fisher Scientific 1:250
Goat anti-Mouse Alexa Fluor 546 A11030 Thermo Fisher Scientific 1:250
Goat anti-Rabbit Alexa Fluor 488 A11034 Thermo Fisher Scientific 1:250
Goat anti-Rat Alexa Fluor 594 A11007 Thermo Fisher Scientific 1:250

Additional Ab used for the 3D Tumour stainings
Donkey anti-Mouse Alexa Fluor 568 A10037 Invitrogen (Thermo Fisher Scientific) 1:1000
Donkey anti-Rabbit Alexa Fluor 647 Invitrogen A-3573 (Thermo Fisher Scientific) 1:1000

Validation

Series of these antibodies have been previously described/validated in other organoid papers: Randriamanantsoa, Papargyriou et al. 2022. Validation statements available from the manufacturers:

Primary:
https://www.sigmaaldrich.com/DE/en/product/sigma/65906
https://www.cellsignal.com/products/antibody-conjugates/e-cadherin-24e10-rabbit-mab-alexa-fluor-488-conjugate/3199

https://www.bdbiosciences.com/en-de/products/reagents/microscopy-imaging-reagents/immunofluorescence-reagents/alexa-fluor-488-mouse-anti-e-cadherin.560061
https://www.cellsignal.com/products/primary-antibodies/n-cadherin-13a9-mouse-mab/14215
https://www.abcam.com/ki67-antibody-ab15580.html
https://www.scbt.com/p/zeb1-antibody-h-102 The ZEB1 antibody (H-102) has been discontinued and replaced by ZEB1 (H-3) : sc-515797.
https://www.cellsignal.com/products/primary-antibodies/yap-antibody/4912
https://www.thermofisher.com/antibody/product/ZO-1-Antibody-clone-ZO1-1A12-Monoclonal/339194
https://www.cellsignal.com/products/primary-antibodies/vimentin-d21h3-xp-rabbit-mab/5741
https://www.bdbiosciences.com/en-de/products/reagents/microscopy-imaging-reagents/immunofluorescence-reagents/purified-mouse-anti-catenin.610153
https://www.cellsignal.com/products/primary-antibodies/pan-keratin-ae1-ae3-mouse-mab/67306
https://www.abcam.com/en-de/products/primary-antibodies/hnf1-alpha-hnf-1b-antibody-epr18644-ab209666
https://www.thermofisher.com/antibody/product/Ki-67-Antibody-clone-SolA15-Monoclonal/14-5698-82
https://www.merckmillipore.com/DE/en/product/Anti-Sox9-Antibody,MM_NF-AB5535
https://www.zytomed-systems.com/assets/datasheets/GA_MSK004_MSK004-05_MSG004_DE_V01.pdf

Secondary:

https://www.thermofisher.com/antibody/product/Goat-anti-Rabbit-IgG-H-L-Highly-Cross-Adsorbed-Secondary-Antibody-Polyclonal/A32732
https://www.thermofisher.com/antibody/product/Donkey-anti-Rabbit-IgG-H-L-Highly-Cross-Adsorbed-Secondary-Antibody-Polyclonal/A10040
https://www.thermofisher.com/antibody/product/Goat-anti-Mouse-IgG-H-L-Highly-Cross-Adsorbed-Secondary-Antibody-Polyclonal/A-11030
https://www.thermofisher.com/antibody/product/Goat-anti-Rabbit-IgG-H-L-Highly-Cross-Adsorbed-Secondary-Antibody-Polyclonal/A-11034
https://www.thermofisher.com/antibody/product/Goat-anti-Rat-IgG-H-L-Cross-Adsorbed-Secondary-Antibody-Polyclonal/A-11007
https://www.thermofisher.com/antibody/product/Donkey-anti-Mouse-IgG-H-L-Highly-Cross-Adsorbed-Secondary-Antibody-Polyclonal/A10037
https://www.thermofisher.com/antibody/product/Donkey-anti-Rabbit-IgG-H-L-Highly-Cross-Adsorbed-Secondary-Antibody-Polyclonal/A-31573

Eukaryotic cell lines

Policy information about cell lines and Sex and Gender in Research

Cell line source(s)	All the murine primary PDAC lines used were previously generated and characterized in Mueller et al 2018. ATCC lines were kindly provided by the lab of Günter Schneider. PDAC PDOs were obtained either from Peschke et al. 2022 or Orben et al 2022. The new lines first described in this study were obtained from the TUM PDO repository (Head Maximilian Reichert). The HEK293T R-spondin-1-overexpressing cell line was provided by the Hubrecht Institute (Uppsalalaan 8, 3584 CT Utrecht, Netherlands).
Authentication	All ATCC lines used were previously authenticated in the lab of Günter Schneider. The HEK293T R-spondin-1 cells were checked for their morphology and used only in early passages.
Mycoplasma contamination	All cell lines tested negative for mycoplasma contaminations.
Commonly misidentified lines (See ICLAC register)	No commonly misidentified cell lines were used.

Animals and other research organisms

Policy information about studies involving animals; ARRIVE guidelines recommended for reporting animal research, and Sex and Gender in Research

Laboratory animals	For the organoid phenotype orthotopic implantation in the syngeneic mouse model, the strains C57Bl/6J (for the line 9591) or B6129SF1/J (for the line 16992) were used. For the orthotopic implantation after pre-treatment with FFX or 8 Gy irradiation, cells were implanted into the pancreas of 8-week-old athymic Crl:NU(NCr)-Foxn1nu mice. All mice were kept in dedicated facilities, enriched housing conditions with a light–dark cycle of 12:12 h, temperature between 20 and 24 °C, and a relative humidity of 55%.
Wild animals	The study did not involve wild animals.
Reporting on sex	Sex was matched to the parental cell line in the syngeneic mouse model. Both ID: 9591-transplanted epithelial organoids and ID: 16992-transplanted mesenchymal organoids were implanted into male animals. For the implantation after pre-treatment, female nude mice were used.
Field-collected samples	This study did not involve samples collected from the field.
Ethics oversight	All animal experiments were performed in accordance with the European guidelines for the care and use of laboratory animals. The animal study was approved by the Institutional Animal Care and Use Committees (IACUC) committee of the Technical University of Munich and the local authorities (Regierung von Oberbayern, Munich, Germany; license 55.2-2532.Vet_02-19-174 for the organoid phenotype transplantation and license 55.2-2532.Vet_02-18-91 for the implantation post FFX or 8 Gy treatment).

Note that full information on the approval of the study protocol must also be provided in the manuscript.
